# Research Progress of Electrochemical Biosensors for Diseases Detection in China: A Review

**DOI:** 10.3390/bios15040231

**Published:** 2025-04-05

**Authors:** Haoran Cui, Xianglin Xin, Jing Su, Shiping Song

**Affiliations:** 1 Institute of Materiobiology, College of Science, Shanghai University, Shanghai 200444, China; haoran@shu.edu.cn (H.C.); xxl23@shu.edu.cn (X.X.); 2 School of Perfume and Aroma Technology, Shanghai Institute of Technology, No. 100 Haiquan Road, Shanghai 201418, China

**Keywords:** electrochemical biosensor, biomarkers, disease diagnosis, China

## Abstract

Disease diagnosis is not only related to individual health but is also a crucial part of public health prevention. Electrochemical biosensors combine the high sensitivity of electrochemical methods with the inherent high selectivity of biological components, offering advantages such as excellent sensitivity, fast response time, and low cost. The generated electrical signals have a linear relationship with the target analyte, allowing for identification and concentration detection. This has become a very attractive technology. This review offers a summary of recent advancements in electrochemical biosensor research for disease diagnosis in China. It systematically categorizes and summarizes biosensors developed in China for detecting cancer, infectious diseases, inflammation, and neurodegenerative disorders. Additionally, the review delves into the fundamental working principles, classifications, materials, preparation techniques, and other critical aspects of electrochemical biosensors. Finally, it addresses the key challenges impeding the advancement of electrochemical biosensors in China and examines promising future directions for their development.

## 1. Introduction

The rapid diagnosis of early-stage diseases is critical for effective treatment, better prognosis, and lower healthcare costs. The optimal treatment window is often missed due to the lack of faster and more efficient diagnostic methods, including cancer, infectious diseases, inflammation, and neurodegenerative disorders. This delay places a devastating financial burden on patients and brings the healthcare system to the brink of collapse [1]. Conventional disease detection methods, such as pathological analysis, imaging examinations, and enzyme-linked immunosorbent assay (ELISA), generally suffer from limitations, including prolonged detection times, high costs, operational complexity, and the inability to perform rapid on-site detection [2]. Therefore, the development of rapid, sensitive, accurate, flexible, non-invasive, and simple diagnostic methods has become a major focus of current research. Electrochemical biosensors can convert physical, chemical, biological, and environmental changes into easily observable signals [3] and have the advantages of rapid detection, simple operation, portable equipment, and low detection costs [4]. For example, label-free electrochemical immunosensors can directly detect changes in the electrochemical signal caused by the binding of antigens to antibodies, eliminating the need for labelling and greatly simplifying the detection process [5]. This offers advantages in terms of low cost, rapid detection speed, and strong anti-interference capabilities, making them suitable for on-site detection [6]. Advances in micromachining manufacturing technologies over the past few decades have facilitated the commercialization of biosensors such as wearables and nanomachines. Currently, the biosensor industry is dominated by electrochemical, paper-based, and optical biosensors [7]. Although these methods still have limitations, such as poor stability of biomaterials, susceptibility to environmental influences, and high cost of mass production, they have been widely used for immediate disease detection, early diagnosis, and chronic disease management due to their real-time and rapid detection capability. They are gradually becoming an important complement to traditional diagnostic methods and may even replace them [8].

Disease biomarkers are measurable and objectively evaluated characteristics that serve as indicators of pharmacological responses to therapeutic interventions, pathogenic processes, or normal biological processes [9]. Historically, biomarkers were physical characteristics or physiological indicators. Today, the term commonly refers to molecular biomarkers [10]. Common biomarkers include proteins, nucleic acids, enzymes, and lipids [11]. When pathological changes occur in the body, the levels of these biomarkers may increase or decrease. Detecting these molecular changes enables disease diagnosis [12]. Biomarkers should possess several common characteristics [13,14]: Biomarkers should exhibit measurable and clinically significant changes in disease progression or treatment; should also have high sensitivity, specificity, and timeliness; and be easily accessible from non-invasive sources, among other requirements.

**Figure 1:**
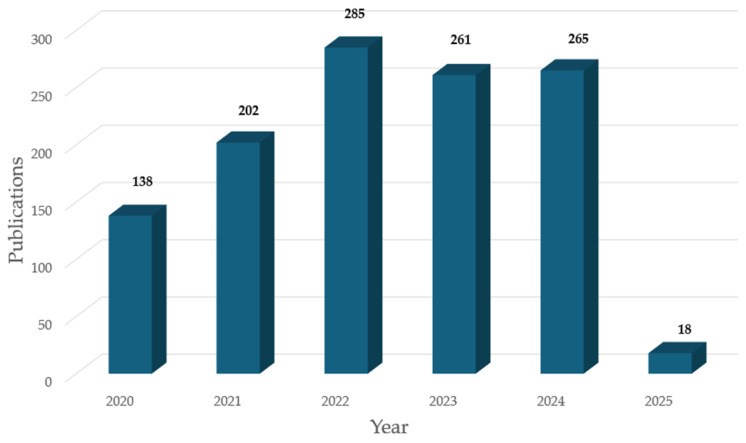
The number of publications on disease-detecting biosensors by Chinese research groups shows increasing research interest, as shown by the number of papers published annually from 2020 to 28 February 2025, as shown in the figure. The results are from Web of Science, using the search terms: topic is “electrochemical biosensor” and “disease diagnosis”. Address is “China”.

**Figure 2:**
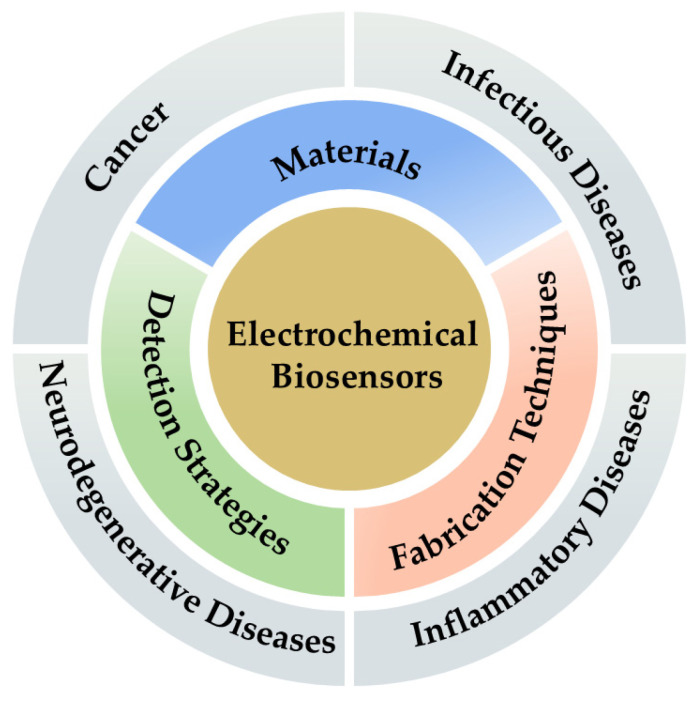
Overview of disease detection with electrochemical biosensors.

Electrochemical biosensors have been extensively investigated for disease diagnostic applications because they are fast, sensitive, and inexpensive. The number of publications on disease-detecting biosensors by Chinese research teams has increased over the past five years (Figure 1). This review summarizes the latest progress made by Chinese researchers in this field over the past five years, covering a variety of disease detection as well as a variety of materials and strategies to improve the performance of the sensors (Figure 2), and provides insightful comments on these studies, aiming to provide valuable references for researchers and practitioners in related fields. Finally, we provide a prospective outlook on the future development of electrochemical biosensors in China.

## 2. Electrochemical Biosensors

**Figure 3:**
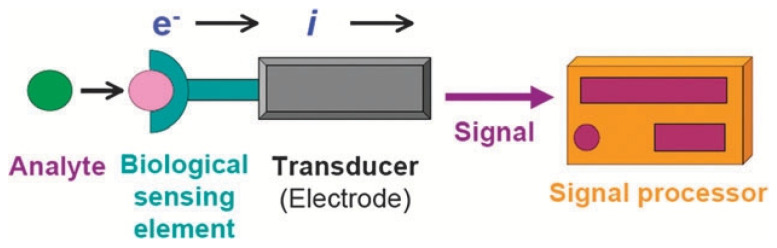
Schematic illustration of the main components of an electrochemical biosensor. Reproduced with permission from [18]. Copyright 2010, Royal Society of Chemistry.

Electrochemical biosensors combine biosensing with electrochemical methods in which the transducer operates based on electrochemical principles, such as changes in voltage, current, conductivity, or resistance (Figure 3). The high specificity of electrochemical biosensors stems from the selective binding of recognition elements to target molecules, minimizing interference from impurities. This strong affinity increases the signal-to-noise ratio, resulting in ultra-low detection limits (typically in the nanomolar or picomolar range), which facilitates the detection of trace analytes with high sensitivity [15]. In electrochemical biosensors, biorecognition events affect the electrochemical behavior of the electrode surface, thereby enabling sensing. Sensing mechanisms include opening access to the electrode surface (OAE), blocking access to the electrode surface (BAE), changing the transport of ions or charge carriers, and modulating the electrostatic affinity or intercalation of the redox probe with different biorecognition probes. Electrochemical biosensors can be categorized based on their signal transduction mechanisms and electrochemical detection methods into the following types: amperometric/voltammetric sensors, potentiometric sensors, conductometric sensors, and impedimetric sensors.

Compared to other sensor technologies, optical biosensors have simple detection methods and can be easily observed by the naked eye without expensive and complex equipment [16]. Paper-based biosensors have the advantages of good biocompatibility, good flexibility, and high cost-effectiveness [17]. Electrochemical biosensors have the advantages of high sensitivity and selectivity. These sensing technologies have their own advantages and can complement each other to achieve multi-signal detection, improve detection accuracy, and develop portable, low-cost sensors for commercial development.

### Principle of Electrochemical Biosensors

Electrochemical biosensors can be classified in terms of the differences between the type of bioreceptor or biological recognition element [19]: enzyme sensors, DNA sensors, immunosensors, and aptamer sensors. The specificity and catalytic activity of enzymes confer high sensitivity and selectivity to these sensors. Furthermore, enzymes can catalyze small-molecule reactions, enabling the rapid detection of low-concentration target molecules. However, enzyme activity is susceptible to external interference, and enzyme extraction and purification are costly. DNA sensors utilize the complementary base pairing between target DNA and its complementary strand. DNA probes can be designed based on the target DNA sequence and, when combined with PCR amplification techniques, can detect target nucleic acids at extremely low concentrations. Immunosensors rely on the highly specific interaction of antigens with antibodies for detection. In high-concentration samples, detection can be performed directly without amplification, making them suitable for high-throughput and cost-effective analysis. However, the performance of immunosensors is limited by extremely high concentrations, short lifespan, and a potential for false positives in complex biological samples. Aptamer sensors represent a novel biosensor technology. They are screened for specific target molecules, exhibiting high specificity in recognition, reducing cross-reactions, and enabling the detection of cells, proteins, and other small molecules. Compared to antibodies, aptamers are easier to prepare, more environmentally stable, and simpler to modify. However, aptamer screening takes longer, and development costs are higher.

## 3. Advances in Materials and Fabrication Technologies for Electrochemical Biosensors

### 3.1. Materials

Advancements in nanoscience and nanotechnology have provided possibilities to enhance electrochemical sensor performance through developing functional nanomaterials. The following is a brief introduction to some of the latest research on materials for disease detection biosensors by a Chinese research group.

#### 3.1.1. Noble Metal Nanomaterials

**Figure 4:**
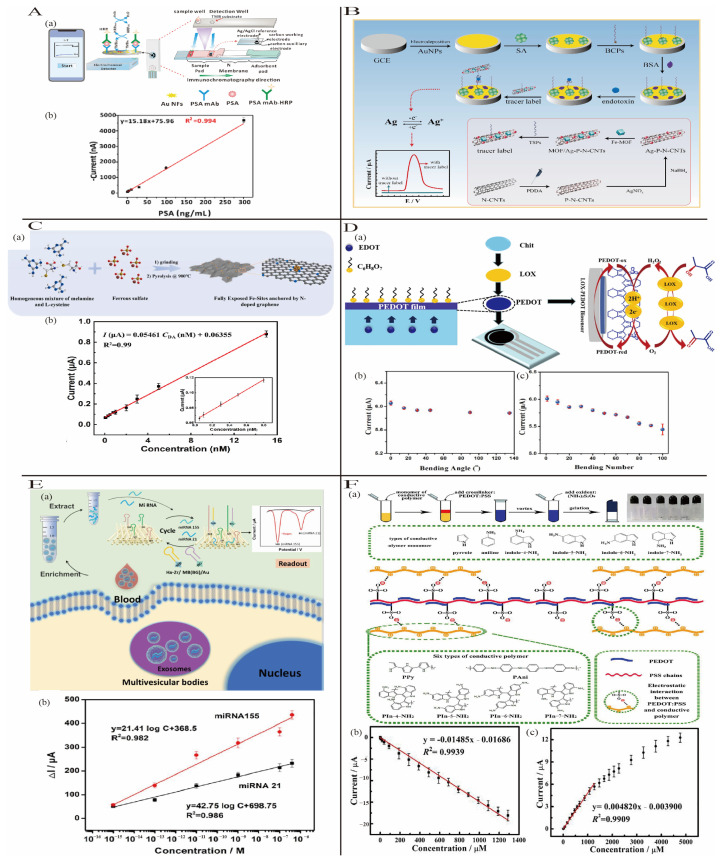
(**A**) (**a**) A schematic diagram of EIC detection chips for PSA detection; (**b**) the linear curve of the PSA response in PBS buffer. Reproduced with permission from [22]. Copyright 2022, MDPI AG. (**B**) The preparation of the proposed electrochemical aptasensor step by step. Reproduced with permission from [24]. Copyright 2022, Elsevier. (**C**) (**a**) Schematic illustration of the synthetic procedure of the Fe/N-GR catalyst; (**b**) calibration curve plotted between DPV response current and exposed DA concentrations. Reproduced with permission from [27]. Copyright 2022, Elsevier. (**D**) (**a**) A schematic diagram of the modified electrode catalytic mechanism; (**b**) the bending angle effect on the PEDOT-2/LOX/SPCE electrode (lactate concentration: 10 mM); (**c**) the bending number effect on the electrode (lactate concentration: 10 mM, bending angle: 135°); reproduced with permission from [29]. Copyright 2022, Elsevier. (**E**) (**a**) The flow diagram of 3D CFP/GWs/Au DNA-circuit test strip; (**b**) linear calibration chart. Reproduced with permission from [31]. Copyright 2022, Elsevier. (**F**) (**a**) Schematic illustration of gelation processes for six types of conductive polymer hydrogels: PPy, PAni, PIn-4-NH_2_, PIn-5-NH_2_, PIn-6-NH_2_, PIn-7-NH_2_; (**b**) the AuNPs/PPy/PEDOT:PSS/GCE dopamine sensor linear calibration curve; (**c**) the linear calibration curve of the PtNPs/PIn-5-NH_2_/PEDOT:PSS/GCE H_2_O_2_ sensor; reproduced with permission from [35]. Copyright 2022, Elsevier.

Noble metal nanomaterials are widely used in electrochemical biosensors because of their large specific surface area, outstanding electrical conductivity, electrocatalytic activity, large surface area, good reaction catalysis, excellent electron transfer capability, and good biocompatibility [20]. The high chemical stability and excellent electrical conductivity of gold nanoparticles (AuNPs) make them the most commonly used noble metal nanomaterials in biosensors. AuNPs are widely employed as signal amplification molecules or carriers for analytical purposes [21]. Song’s group developed an innovative biosensing system based on a gold nanofiber-modified SPCE interface for PSA detection [22]. The presence of Au NFs significantly enhanced electron transfer efficiency, further improving the device’s sensitivity (Figure 4A). This biosensor achieved a linear detection range of 0 to 100 ng/mL with a low detection limit (LOD) of 0.28 ng/mL (8.78 fM).

Compared to AuNPs, silver nanoparticles (AgNPs) exhibit high oxidative activity, enabling strong interactions with target molecules, and exhibit excellent reactivity in electrochemical processes. They are commonly employed for amplifying electrochemical signals by enhancing redox currents [23]. In order to achieve ultrasensitive endotoxin detection, Mu et al. [24] used a sandwich-type electrochemical aptasensor, utilizing Metal–Organic Frameworks (MOF)/Ag-P-N-CNT nanohybrids decorated with AgNPs. The AgNPs not only immobilized thiol-modified signal probes (TSPs) but also provided exceptional electrochemical activity, significantly enhancing signal amplification (Figure 4B). This sensor exhibited a broad detection range (1 fg/mL to 100 ng/mL), with a detection limit of 0.55 fg/mL.

#### 3.1.2. Carbon-Based Nanomaterials

Carbon-based nanomaterials are a type of low-dimensional material. Graphene and carbon nanotubes, primarily composed of sp^2^ carbon atoms, form a conjugated π–electron network, granting them exceptional electrical conductivity and mechanical properties [25]. Additionally, doping carbon nanomaterials with foreign atoms can further modulate their electrochemical sensing characteristics, significantly enhancing their functionality and application potential [26]. Sun et al. [27] addressed the insufficient exposure of active Fe sites in Fe-based nanomaterials by designing an electrochemical biosensor for dopamine (DA) detection based on Fe/N-doped graphene (Fe/N-GR). The low-layer N-doped graphene improved electron transfer efficiency while providing abundant functional groups to enhance Fe active site exposure and interactions with DA (Figure 4C). This sensor achieved a linear relationship in a wide range of 50 pM–15 nM, and the detection limit of 27 pM was ultra-low.

#### 3.1.3. Conductive Polymer-Based Nanomaterials

Conductive polymers (CPs) are organic macromolecular materials composed of conjugated chemical double bonds on polymer chains. They not only have excellent conductivity but also have the unique bending resistance and stretchability of polymers, making them indispensable in the preparation of wearable biosensors [28]. Zhu et al. [29] synthesized large-area, ordered poly(3,4-ethylenedioxythiophene) (PEDOT) films by using an interfacial synthesis method and deposited them onto screen-printed carbon electrodes (SPCEs). Subsequently, lactate oxidase (LOX) was immobilized onto the PEDOT film via cross-linking and adsorption, forming a flexible sensor for sweat lactate detection (Figure 4D). The sensor exhibited a detection limit of 0.083 mmol/L and maintained excellent stability.

#### 3.1.4. Porous Materials

Metal–organic frameworks (MOFs) and covalent organic frameworks (COFs) are two rapidly growing classes of crystalline materials. Their exceptional adsorption properties, high porosity, and tunable surface functionalities provide an ideal environment for anchoring biomolecular recognition elements [30]. Yang et al. [31] used nucleic acid-functionalized zirconium MOFs (Zr-MOFs) to develop a disposable paper-based electrochemical sensing platform for the simultaneous label-free and enzyme-free detection of exosomal miRNA-155 and miRNA-21 (Figure 4E). Specific recognition of different miRNAs was achieved by modifying the dye loading and hairpin DNA structure on Zr-MOFs. The detection limits for exosomal miRNA-155 and miRNA-21 were as low as 33.4 aM and 23.1 aM, respectively.

Biochar is a porous carbonaceous solid material generated by partial oxidation of organic sources, such as animals or plants, with a rich pore structure, high carbon content, and strong adsorption capacity [32,33]. Yang et al. [34] constructed an electrochemical biosensor consisting of Biochar-loaded Cu^2+^/Cu^+^ composites for the detection of ractopamine. Biochar with a three-dimensional porous network structure effectively improved the sensitivity of the device.

#### 3.1.5. Gel Materials

Gels are three-dimensional network structures formed through chemical cross-linking or physical entanglement, capable of retaining large amounts of liquid without dissolving. Therefore, gels combine the outstanding mechanical properties of solid materials with the high ionic conductivity of liquid electrolytes [36]. Composite gel materials have significantly improved mechanical performance, biocompatibility, and electrochemical activity, expanding their applications in electrochemical biosensors [37]. Yang et al. [35] explored a general method to synthesize conductive polymer/PEDOT: PSS hydrogels by leveraging the electrostatic interactions between negatively charged SO_3_^−^ groups in PEDOT: PSS and positively charged polymer chains of polyaniline (PAni), polypyrrole (PPy), and poly(aminoindole) (PIn-X-NH_2_) (Figure 4F). This approach dramatically enhanced the conductivity (increased by 10–15 times) and biocompatibility of the hydrogels. An electrochemical biosensor based on these conductive polymers/PEDOT: PSS hydrogels was fabricated and achieved detection limits of 6.7 nM and 0.67 μM for dopamine and hydrogen peroxide, respectively

### 3.2. Fabrication Techniques for Electrochemical Biosensors

The structural design of electrochemical biosensors is closely related to the properties of the analyte, the working environment of the sensor, and the required detection parameters [38].

#### 3.2.1. Printing Methods

**Figure 5:**
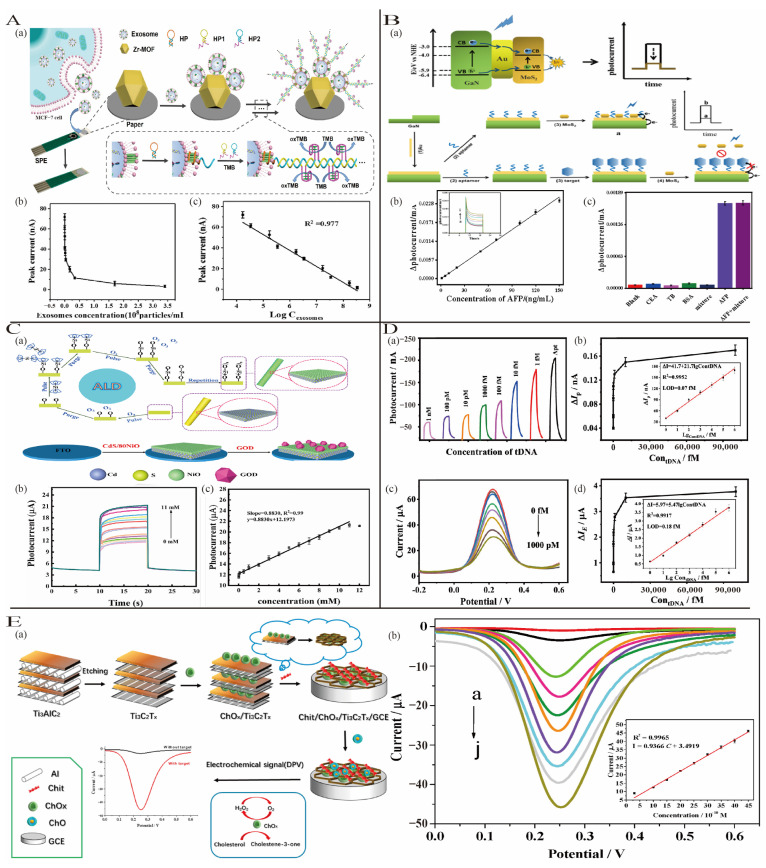
(**A**) (**a**) The paper-based exosome assay biosensor principle; (**b**) the relationship between the intensity of the DPV peak current and the concentration of exosomes; (**c**) the linear relationship between the peak intensity of the current and the logarithm of the concentration of the exosomes; reproduced with permission from [40]. Copyright 2021, American Chemical Society. (**B**) (**a**) Charge-transfer mechanism in MoS_2_/Au/GaN; (**b**) linear relationship between the difference of the photocurrent in the presence and absence of AFP (ΔI) and AFP concentration in the range of 1.0–150 ng/mL; (**c**) the AFP detection specificity of the PEC sensor; reproduced with permission from [42]. Copyright 2021, American Chemical Society. (**C**) The production of precisely tuned CdS/xNiO nanoscaled materials via ALD and label-free PEC biosensor-based CdS/80NiO-GOD (**a**); PEC signal (**b**) and calibration curve; (**c**) of CdS/80NiO-GOD biosensor at different glucose concentrations; reproduced with permission from [43]. Copyright 2022, Elsevier. (**D**) (**a**) Photocurrent responses; (**b**) the PEC biosensor calibration curve; (**c**) DPV plots using the Cu-MOF@CuPc-TA-COF-based biosensor for the detection of tDNA at different concentrations; (**d**) calibration curves between ΔIE and the concentration of tDNA; reproduced with permission from [44]. Copyright 2021, American Chemical Society. (**E**) (**a**) Schematic representation of the fabrication of the Chit/ChOx/Ti_3_C_2_T_x_/GCE and a possible reaction mechanism of cholesterol at the modified GCE; (**b**) Chit/ChOx/Ti_3_C_2_T_x_/GCE biosensor DPV at different cholesterol concentrations. Reproduced with permission from [44]. Copyright 2021, Elsevier.

Printed electronics combine high-performance functional material inks with various printing techniques to enable the efficient production of large-area flexible electronic devices to meet the diverse demands of modern applications. Printing techniques can be broadly classified into contact-based methods (e.g., screen printing, gravure printing, and nanoimprint lithography) and non-contact methods (e.g., inkjet printing, electron beam evaporation, and aerosol jet printing) [39]. Li et al. [40] developed a Zr-MOF-functionalized paper-based electrochemical biosensor integrated with a screen-printed electrode (SPE) for ultrasensitive tumor-derived exosome detection. In this sensor, Zr-MOFs and aptamers served as the recognition system, while the signal was amplificated by hybridization chain reaction (HCR) and DNAzyme-mediated catalysis (Figure 5A). The SPE not only acted as a carrier for functional materials but also provided economical and portable features for the sensor. The sensor had a detection range of 1.7 × 10^4^ to 3.4 × 10^8^ particles/mL and a low detection limit (5 × 10^3^ particles/mL).

#### 3.2.2. Vapor Deposition Techniques

PVD employs physical processes such as evaporation, ionization, and sputtering to vaporize materials and deposit them onto a substrate surface. The primary techniques include vacuum evaporation, magnetron sputtering, ion plating, and arc evaporation [41]. Wang et al. [42] utilized direct current sputtering to deposit a uniform Au film on GaN, forming an Au/GaN working electrode. They developed a MoS_2_/Au/GaN-based photoelectrochemical (PEC) aptasensor to detect the cancer biomarker alpha-fetoprotein (AFP). When AFP binds to the aptamer, steric hindrance prevents MoS_2_ from contacting the Au/GaN electrode, reducing photocurrent suppression and resulting in a significant enhancement of the signal (Figure 5B). This sensor has a detection limit of 0.3 ng/mL and excellent selectivity for AFP.

CVD deposits solid materials onto a substrate surface through gas-phase chemical reactions and is currently the most effective method for low-cost, large-scale preparation of two-dimensional materials. Due to its versatility in depositing a wide range of materials, CVD has seen rapid development. Zhang et al. [43] developed a photoelectrochemical biosensor for glucose detection based on CdS/80NiO nanosemiconductor materials and the highly specific and high-affinity glucose oxidase (GOD). A complete p-n heterojunction of CdS/80NiO was synthesized using different ALD cycle numbers, maintaining the high light absorption efficiency of CdS (Figure 5C). The sensor demonstrated an exceptionally low detection limit of 3.4 × 10^−10^ M, showcasing excellent sensitivity and selectivity.

#### 3.2.3. Template Synthesis and Self-Assembly

Template synthesis is a relatively simple and straightforward process that allows for the direct growth of new materials [45]. By utilizing a pre-designed template, materials with unique structures, morphologies, and properties can be synthesized, followed by the removal of the template. Xu et al. [44] developed a MOF@COF heterostructure composite using a template synthesis method to construct photoelectrochemical and electrochemical biosensors for the detection of HIV-1 DNA. Copper-based MOF (Cu-MOF) was used as the template, on which a covalent organic framework (COF) was grown in situ, resulting in a heterostructure with enhanced photoelectrochemical and electrochemical activity (Figure 5D). This template synthesis strategy enabled the composite to achieve an ultralow detection limit of 0.07 fM for HIV-1 DNA using a dual-mode photoelectrochemical–electrochemical sensor, demonstrating significant potential for applications in the field of biosensing.

Self-assembly is a process by which molecular units spontaneously organize into ordered structures through non-covalent or weak covalent interactions under thermodynamic and kinetic conditions, including π-π stacking, hydrogen bonding, van der Waals forces, and electrostatic and hydrophobic interactions [46]. Hou et al. [47] developed a sensitive and straightforward enzyme-based electrochemical biosensor using MXene (Ti_3_C_2_T_X_) for cholesterol detection (Figure 5E). Ti_3_C_2_T_X_ nanosheets served as templates for immobilizing cholesterol oxidase (ChO_x_). Through a sequential self-assembly process, chitosan (Chit) was electrostatically bound to Ti_3_C_2_T_X_, enabling the uniform fixation of ChO_x_ on the Ti_3_C_2_T_X_ surface. This process yielded a layered, accordion-like Chit/ChOx/Ti_3_C_2_T_X_ nanocomposite, providing favorable conditions for the biomolecular transfer and enhanced electrical signal amplification. The biosensor achieved a linear range of 0.3 to 4.5 nM for cholesterol concentrations detection; the detection limit was 0.11 nM, and the sensitivity was 132.66 μA nM^−1^ cm^−2^.

### 3.3. Detection Strategies for Enhancing the Performance of Electrochemical Biosensors

The development of nanoscience and micro/nanofabrication technologies have significantly advanced the preparation techniques for electrochemical biosensors, driving their evolution toward miniaturization, integration, and enhanced selectivity and sensitivity.

#### 3.3.1. Strategy Based on Nucleic Acid

**Figure 6:**
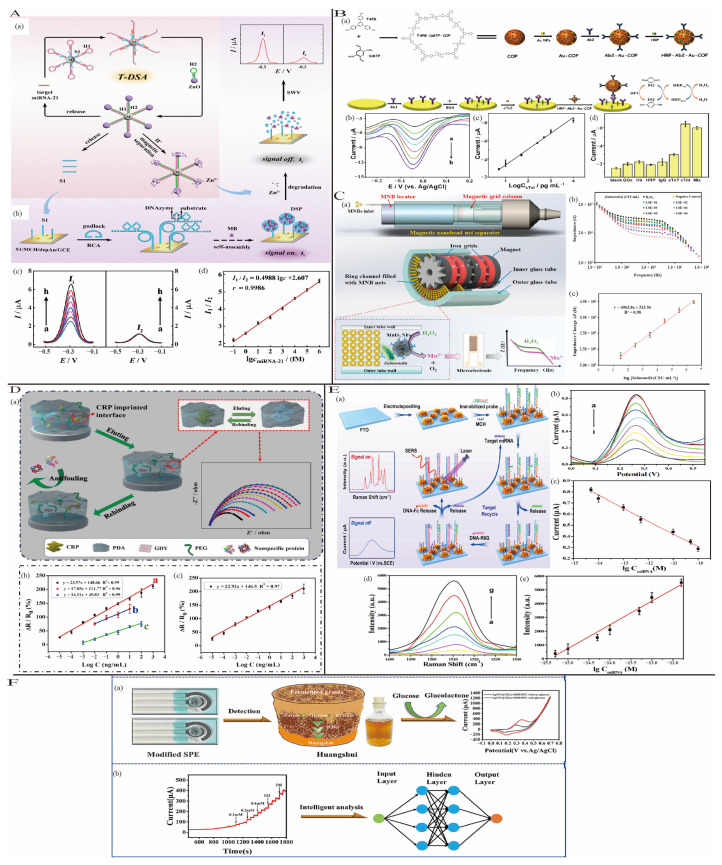
(**A**) The ratiometric electrochemical biosensor principle: (**a**) target-driven dual-signal amplification and (**b**) the method of modifying the electrode interface and the signal response; (**c**) biosensor SWV curves with different miRNA-21 concentrations; (**d**) the linear relationship between the ratio of I_1_/I_2_ and the logarithm of miRNA-21 concentration; reproduced with permission from [49]. Copyright 2022, American Chemical Society. (**B**) (**a**) A schematic showing the synthesis of HRP-Ab2-Au-COF and the construction of the biosensor to detect cTnI; (**b**) DPV response curves of the presented electrochemical immunosensor (concentration of cTnI from a to h: 0–10 ng/mL) and (**c**) the linear relationship between the current and the logarithm of cTnI concentration; (**d**) selectivity at 1 ng/mL of cTnI and other interfering proteins (10 ng/mL); reproduced with permission from [56]. Copyright 2021, American Chemical Society. (**C**) (**a**) A diagram of this impedance biosensor for the detection of Salmonella; (**b**) electrochemical impedance spectra of Salmonella at the concentrations of 3.0 × 10^1^–3.0 × 10^6^ CFU/mL in the pure cultures; (**c**) calibration curve of this biosensor for detection of Salmonella at the concentrations of 3.0 × 10^1^–3.0 × 10^6^ CFU/mL (*n* = 3); reproduced with permission from [59]. Copyright 2021, Elsevier. (**D**) (**a**) A GDY-based CRP-imprinted biosensor schematic; (**b**) calibration curves of the CRP-imprinted biosensor towards target CRP in the absence ((**a**): C-MIPs biosensor; (**b**): CRP-imprinted PDA biosensor; (**c**) CRP-imprinted PDA/PEG biosensor) and presence (**c**) of 10% (*v/v*) FBS. Reproduced with permission from [60]. Copyright 2022, Elsevier. (**E**) (**a**) A schematic illustration of the proposed strategy for the enzyme-free detection of the amplification of miRNA by target recycling; (**b**) typical DPV responses of the sensors to different concentrations of the target miRNA-21 and (**c**) corresponding calibration plot for the DPV peak current; (**d**) Raman Detection of miRNA-21 and (**e**) corresponding linear fitting curve of peak intensity changes for (**d**); reproduced with permission from [61]. Copyright 2021, American Chemical Society. (**F**) (a) The employment of AgNPs@NiCo-MOF/SPE in the detection of glucose in Huangshui; (**b**) the machine learning mode for the intelligent analysis of sensor detection data; reproduced with permission from [62]. Copyright 2023, Elsevier.

DNA amplification techniques significantly enhance the original signal by repeatedly replicating target DNA sequences, thereby improving the sensitivity of sensors. These techniques include Rolling-Circle Amplification (RCA), Hybridization Chain Reaction (HCR), and Loop-Mediated Isothermal Amplification (LAMP) [48]. Kong et al. [49] proposed a ratiometric electrochemical biosensor based on a target-induced dual signal amplification (T-DSA) strategy for ultrasensitive detection of miRNA-21 (Figure 6A). This method utilizes target miRNA-21 in a small amount to trigger amplification and produce a large quantity of mimic DNA S1 and Zn^2+^ through acid dissolution. DNA S1 initiates RCA to generate functionalized DNA nanospheres (DSP) in situ on the electrode, effectively capturing a significant amount of signal tag methylene blue (MB) and producing a strong electrochemical signal (signal on, I_1_). Simultaneously, Zn^2+^ acts as a cofactor to trigger DNAzyme-catalyzed cleavage of DSP, releasing MB and reducing the electrochemical response (signal off, I_2_). With a detection limit as low as 26.7 aM, the quantification detection of miRNA-21 was achieved by measuring the I_1_/I_2_ ratio.

DNA nanotechnology has attracted considerable attention because of its inherent self-assembly properties, high controllability, and programmability, with structures such as DNA nanorods, DNA nanowires, and DNA tetrahedra. Among these, DNA tetrahedral nanostructures consist of four single-stranded nucleic acids that self-assemble into a three-dimensional tetrahedral shape based on complementary pairing. The vertices of the DNA tetrahedron are typically formed by the 5′- or 3′-ends of the edge strands, which can be modified through methods such as phosphorylation, amination, or thiolation [50]. Su et al. [51] proposed an electrochemical biosensor functionalized with tetrahedral DNA structure probes (TSP) on a screen-printed carbon electrode (SPCE) for the highly sensitive detection of various bioactive molecules. The DNA tetrahedron provided a rigid support structure, optimizing probe orientation and binding efficiency while reducing nonspecific adsorption. By employing covalent coupling, the TSP was stably immobilized on the SPCE, significantly enhancing the signal-to-noise ratio (S/N ratio) and detection sensitivity. The sensor was successfully applied to detect miRNA-141, proteins (e.g., thrombin), and small molecules (e.g., ATP), achieving a detection limit as low as 10 aM.

DNA walkers are dynamic DNA nanodevices that can move along pre-designed DNA chains or surface tracks. Through dynamic movement, DNA walkers trigger multi-step reactions and ultimately amplify signal output. Compared with DNA amplification technology, DNA walkers focus on the precise control of the movement of DNA molecules at the nanoscale, not simply DNA replication [52]. Miao et al. [53]. proposed a bipedal DNA walker based on a dumbbell-to-wheel structural transformation for ultrasensitive nucleic acid detection. The DNA wheel walker relies on DNAzyme-catalyzed cleavage reactions to dynamically move along the electrode surface, sequentially cutting probes and releasing electrochemical signal molecules, leading to signal reduction. By integrating a pH-controllable DNA triplex for regeneration, the electrode becomes reusable. This method achieved a detection limit as low as 2.2 aM and is applicable to various targets.

#### 3.3.2. Strategy Based on Enzyme

Enzymes are extensively utilized in electrochemical detection due to their high catalytic activity and substrate specificity. They can amplify electrochemical signals not only by participating in nucleic acid amplification but also by catalyzing enzymatic substrates to generate signal molecules for signal amplification [54]. Commonly used enzyme labels include horseradish peroxidase (HRP), alkaline phosphatase (ALP), and glucose oxidase (GO_x_) [55]. Yang et al. [56] proposed an electrochemical immunosensor for detecting acute myocardial infarction (AMI) biomarker cardiac troponin I (cTnI) based on an enzyme-catalyzed signal amplification strategy (Figure 6B). Upon binding the target cTnI to the sensor, HRP catalyzed the oxidation of hydroquinone (HQ) to benzoquinone (BQ) in the presence of H_2_O_2_. Subsequently, BQ was reduced back to HQ at the electrode surface, re-entering the oxidation process and enabling cyclic amplification of the electrochemical signal. This sensor, profiting from the H_2_O_2_-HRP-HQ signal amplification module, showed a good linear relationship over the range of 5 pg/mL to 10 ng/mL, with a detection limit as low as 1.7 pg/mL.

#### 3.3.3. Strategy Based on Magnetic Nanoparticles

Magnetic nanoparticles (MNPs) are a type of nanomaterial characterized by small particle size, large surface area, magnetic responsiveness, and ease of surface modification [55]. On the one hand, as nanoparticles, MNPs provide a high surface area, enabling the immobilization of large quantities of biomolecules, thereby lowering the detection limit [57]. Additionally, by using external magnets, MNPs specifically modified for target analytes can achieve separation and purification, enhancing sensor selectivity in complex environments [58]. Xue et al. [59] developed an impedance biosensor for the rapid and sensitive detection of Salmonella, utilizing a multiloop magnetic nanobead network and manganese dioxide nanoflowers (MnO_2_ NFs) in a loop channel. Magnetic nanobeads (MNBs) modified with capture antibodies (CAbs) specific to Salmonella formed a multilayer loop network under a high-gradient magnetic field, enabling the separation of target bacteria from large-volume samples (10 mL) within a short time (~20 min) (Figure 6C). MnO_2_ NFs effectively amplified impedance signals, enhancing the sensitivity of the biosensor. The sensor achieved a detection limit of 19 CFU/mL and a linear range of 3.0 × 10^1^ to 3.0 × 10^6^ CFU/mL.

#### 3.3.4. Strategy Based on Molecular Imprinting Technology (MIP)

Based on the “lock-and-key” principle, molecular imprinting is a recognition method that uses artificial/synthetic molecules, where the tailored building blocks correspond to the shape, size, and functional groups of the template molecule [63,64]. It can be broadly categorized into two types: host-guest chemistry and self-assembly. Cui et al. [60] proposed an electrochemical biosensor based on protein molecularly imprinted polymers (MIPs) modified with graphdiyne (GDY) nanosheets for detecting human C-reactive protein (CRP). The protein MIPs, formed by polymerizing CRP template molecules with dopamine (DA) as the functional monomer, exhibited excellent selectivity. The sensor demonstrated excellent linearity for CRP detection within the range of 10^−5^ to 10^3^ ng/mL, with a detection limit as low as 0.41 × 10^−5^ ng/mL (Figure 6D).

#### 3.3.5. Strategy Based on Multi-Modal/Signal Synergy

The integration of two or more modes/signals allows a single sensing platform to gather more information, enabling highly sensitive and selective detection of multiple analytes, thereby significantly enhancing diagnostic accuracy [65]. Xu et al. [61] developed a dual-mode biosensor for ultrasensitive detection of miRNA by combining three-dimensional (3D) popcorn-shaped gold nanofilms with toehold-mediated strand displacement reactions (TSDRs). The biosensor demonstrated detection limits of 0.12 fM in the SERS mode and 2.2 fM in the electrochemical mode, with high selectivity and low background noise (Figure 6E).

#### 3.3.6. Strategy Based on Machine Learning

Conceptually, machine learning (ML) is an automated approach to data analysis that trains computers to recognize patterns and make predictions or decisions based on algorithms and statistical models [66]. ML can improve the selectivity and accuracy of biosensors by classifying different substances with approximate response curves through a deep-learning algorithm [67,68]. Hou et al. [62] developed a non-enzymatic electrochemical glucose sensor based on AgNP-modified flower-like bimetallic metal–organic framework (NiCo-MOF) composites, and an artificial neural network (ANN) machine learning model was integrated with this sensor for glucose detection in hydrolysate (HS). The sensor exhibited a fast response time (4 s) and a high sensitivity of 1191.84 μA mM^−1^ cm^−2^ (Figure 6F). Additionally, with the ANN model, the linear detection range was extended to 11 mM with sensor-based intelligent data analysis and prediction. This approach introduces a new method for intelligent analysis in electrochemical sensors.

## 4. Electrochemical Biosensors for Diseases Detection

### 4.1. Cancer

Cancer is one of the number-one causes of death in the world. According to reports, there were nearly 20 million new cancer cases in the world in 2022, and the number of cancer deaths reached 9.7 million, making it the second leading cause of death in the world [69,70]. Cancer is characterized by high incidence, high recurrence rates, and potential lethality. Cancer biomarkers, which indicate the presence of cancer cells in the body, provide critical information for early detection, diagnosis, and treatment. Common cancer biomarkers, such as prostate-specific antigen (PSA), carcinoembryonic antigen (CEA), CA 125, CA 19-9, and alpha-fetoprotein (AFP) [71], have been extensively studied for early cancer screening, diagnosis, and monitoring. The following sections will categorize different types of cancer and highlight the latest advancements in cancer detection using electrochemical biosensors in China.

#### 4.1.1. Leukemia

Leukemia is a group of malignant diseases originating from hematopoietic stem cells, whose abnormal white blood cells uncontrollably proliferate. The abnormal white blood cells lose their ability to differentiate and mature. These abnormal white blood cells occupy the bone marrow and other hematopoietic organs, suppressing normal hematopoiesis and resulting in reduced numbers of white blood cells, red blood cells, and platelets. This ultimately leads to a series of symptoms, including anemia, bleeding, and infections [72]. Among leukemia subtypes, acute promyelocytic leukemia (APL) is a variant of acute myeloid leukemia (AML) and is typically caused by a translocation between chromosomes 15 and 17, resulting in the PML-RARα gene fusion. This abnormal gene disrupts myeloid cell differentiation, leading to leukemia [73]. Zhang et al. [74], utilizing carbon dots/graphene oxide (CDs/GO) nanocomposites, developed an electrochemical DNA biosensor for detecting the PML-RARα fusion gene. The sensor combines the high conductivity of carbon dots with the large surface area and stable binding sites of graphene oxide. It uses the specific hybridization reaction between a gene probe and target DNA to detect APL through changes in electrochemical signals. The sensor has a linear range from 2.5 × 10^−10^ M to 2.25 × 10^−9^ M and a detection limit of 83 pM (S/N = 3), demonstrating excellent sensitivity and specificity.

**Figure 7:**
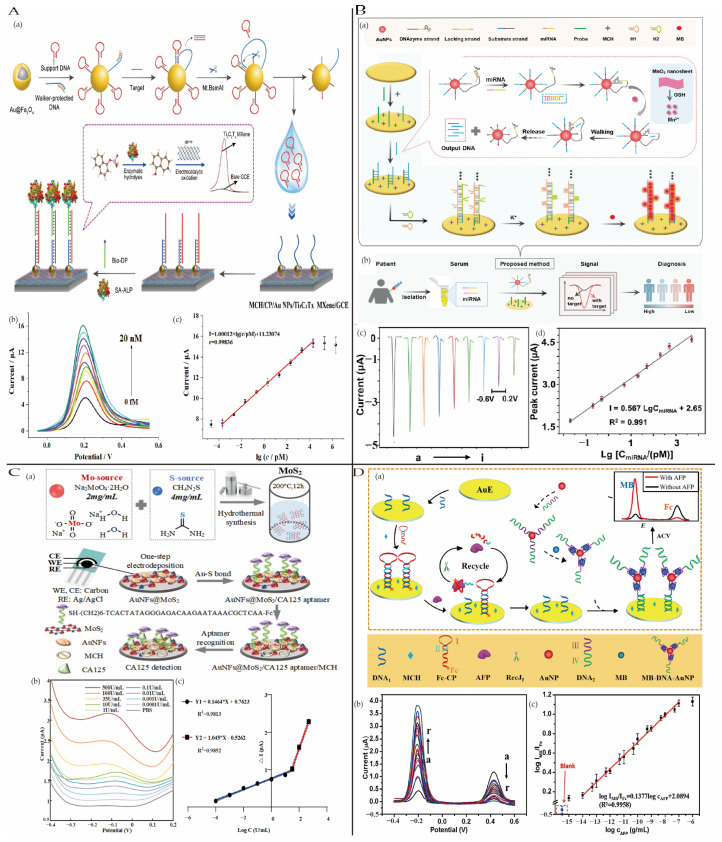
(**A**) (**a**) Schematic representation of the operating principle of the electrochemical biosensor; (**b**) the DPV curves of the electrochemistry sensor with increasing concentrations of the target BCR/ABL fusion gene and (**c**) the corresponding linear relationship between DPV signal and logarithmic value of target BCR/ABL fusion gene concentrations; reproduced with permission from [76]. Copyright 2022, WILEY VCH. (**B**) The electrochemical miRNA assay strategy principle. (**a**) DNAzyme-cleavage cycling amplification and hybridization chain reaction (HCR) amplification; (**b**) the workflow for detecting miRNA in patient serum; (**c**) the results of the LSV for the analysis of miRNA at different levels of concentration; (**d**) the linear relationship between the absolute value of the LSV peak current and the logarithm of the miR-486-5p concentration; reproduced with permission from [81]. Copyright 2022, John Wiley and Sons. (**C**) Schematic diagrams of the sensing platform. (**a**) The synthesis of MoS_2_ and modification of AuNFs@MoS_2_/CA125 aptamer/MCH for CA125 detection; (**b**) DPV curves of CA125 detection from 0.0001U/mL to 500 U/mL in PBS; (**c**) fitting curves between the concentration and ΔI (ΔI = I_CA125-IPBS_). Reproduced with permission from [88]. Copyright 2023, Elsevier. (**D**) (**a**) A schematic representation of the ratiometric electrochemical detection of AFP; (**b**) the ACV responses of the aptasensor to different concentrations of AFP; (**c**) linear relationship between logarithm of I_MB_/I_Fc_ and logarithm of AFP concentration; reproduced with permission from [89]. Copyright 2020, Elsevier.

Chronic myeloid leukemia (CML), also known as chronic myelogenous leukemia, is a clonal hematopoietic stem cell disorder characterized by the translocation of the ABL1 gene on chromosome 9 and the BCR gene on chromosome 22, forming the BCR-ABL1 fusion gene. This fusion gene produces an abnormal protein (tyrosine kinase) that stimulates uncontrolled white blood cell growth [75]. Yu et al. [76] developed an electrochemical biosensor based on Ti_3_C_2_T_x_ MXene nanozymes for detecting the BCR/ABL fusion gene. The sensor employs a cascade catalytic strategy: alkaline phosphatase (ALP) converts 1-naphthyl phosphate (1-NPP) into 1-naphthol, which is subsequently oxidized by Ti_3_C_2_T_x_ MXene to enhance the electrochemical signal. Combined with a DNA walker, the sensor achieves target recognition and multistage signal amplification. It exhibits a linear detection range from 0.2 fM to 20 nM and an ultra-low detection limit of 0.05 fM (S/N = 3) (Figure 7A). In serum samples, the detection results are highly consistent with RT-PCR.

Additionally, Yang et al. [77] developed a split-type electrochemical biosensor (S-E-ELDM) based on magnetic beads and enzyme-labeled DNA for highly sensitive detection of the BCR/ABL^p210^ fusion gene. The sensor utilizes magnetic separation to decouple the target recognition event from electrochemical measurements, minimizing interference from complex matrices. Loop-mediated chain reaction (LCR) enables exponential signal amplification of the target DNA. The sensor achieves a detection limit of 1 aM and, through the integration of dual LCR and OR logic gate designs, successfully detects both e13a2 and e14a2 isoforms simultaneously.

#### 4.1.2. Lung Cancer

The latest estimates from the International Agency for Research on Cancer (IARC) reveal that lung cancer was the most diagnosed cancer in 2022, accounting for an estimated 2.5 million new cases (12.4% of global cancer diagnoses). With an estimated 1.8 million deaths (18.7%), it is also the leading cause of cancer-related deaths. In most countries, the five-year survival rate for lung cancer is often below 20% [69]. Among patients diagnosed at an early stage, nearly 60% survive for five years post-diagnosis, while only 6.3% of those diagnosed at an advanced stage survive beyond five years [78]. However, more than half of lung cancer cases are diagnosed at a late stage, contributing to the low survival rates [79]. Some biomarkers, including carcinoembryonic antigen (CEA), epithelial cell adhesion molecule (EpCAM), circulating microRNAs (miRNAs), and circulating tumor DNA (ctDNA), can be used for lung cancer detection [80]. Meng et al. [81] developed an integrated electrochemical platform for miRNA detection based on DNAzyme cleavage cycling amplification and HCR amplification. Glutathione reduces MnO_2_ nanosheets to Mn^2+^, activating the DNAzyme cleavage cycle to produce a large number of output DNA strands (Figure 7B). These strands trigger HCR to form G-quadruplex/dsDNA structures, which embed methylene blue (MB), significantly enhancing the electrochemical signal. The sensor achieved a detection limit of 5.68 fM for miRNA, with a linear range from 20 fM to 5 nM.

CYFRA21-1, a cytokeratin 19 fragment located in the cytoskeleton of epithelial cells, is considered an effective tumor biomarker for detecting lung cancer. Wang et al. [82] reported a sandwich-type electrochemiluminescence (ECL) biosensor based on CD-MOF@Ru(bpy)_3_^2+^ nanocomposites for detecting CYFRA21-1, a tumor biomarker of non-small cell lung cancer (NSCLC). The combination of cyclodextrin-based MOFs (CD-MOFs), which possess a large surface area and excellent biocompatibility, with Ru(bpy)_3_^2+^, known for its outstanding ECL properties, significantly enhanced the sensor’s detection sensitivity and stability. The biosensor exhibited high sensitivity and a low detection limit of 0.006 ng/mL for CYFRA21-1, along with a good linear range of 0.1–50 ng/mL.

#### 4.1.3. Ovarian Cancer

Ovarian cancer (OC) is the most lethal malignancy in gynecological tumors as well as the seventh most common malignancy worldwide. According to the latest statistics on ovarian cancer in the United States for 2024, there are an estimated 19,680 new cases and 12,740 expected deaths, with a five-year relative survival rate of 51% [83]. OC is often diagnosed at an advanced stage, leading to a poor prognosis, and it has the highest mortality rate among gynecological cancers [84,85]. Some biomarkers, including CA125, HE4, CA19-9, and miRNA, can be used for ovarian cancer detection [86]. Xu et al. [87] fabricated a sandwich-type electrochemical immunosensor for the quantitative analysis of HE4, utilizing PB as a signal indicator and TiMOF-KB@AuN as an efficient signal amplifier. The modification with KB and TiMOF enhanced the conductivity and surface area of the materials, providing more immobilization sites for specific antibodies and accelerating electron transfer at the electrode surface. This significantly improved the sensitivity and response speed of the sensor. The sensor exhibited a wide linear range (0.1–80 ng/mL) and a low detection limit (0.02 ng/mL). It also demonstrated good selectivity, reproducibility, and stability. Additionally, in spiked recovery tests using real samples, the recovery rates ranged from 97.10% to 114.07%, with an RSD of less than 8.64%, indicating excellent practical application potential. Furthermore, Hu et al. [88] prepared an electrochemical aptasensor based on AuNFs@MoS_2_ nanocomposites for the detection of the ovarian cancer biomarker CA125. The AuNFs@MoS_2_-modified electrode was prepared through one-step electrodeposition, providing a large surface area and thiol binding sites as the sensing interface, which significantly enhanced the sensor’s electrochemical performance. The CA125 aptamer offered specific recognition, and MCH blocking was employed to reduce nonspecific adsorption (Figure 7C). The sensor achieved an ultralow detection limit of 0.0001 U/mL, far below the levels found in healthy individuals.

#### 4.1.4. Other Cancers

Colorectal cancer (CRC), encompassing both colon and rectal cancer, is one of the most common malignancies worldwide. According to recent statistics, over 1.9 million new CRC cases (including anal cancer) and 904,000 deaths were reported in 2022, accounting for nearly one-tenth of all cancer cases and deaths [69]. Globally, CRC ranks third in incidence but second in mortality [90]. Studies have shown that early screening and monitoring can significantly reduce CRC incidence and mortality while substantially lowering treatment costs for patients [91]. Only exosomes secreted by tumor cells containing various key bioactive molecules can be used for qualitative and quantitative detection of tumor cells. Wang et al. [92] designed an electrochemical sensor based on functionalized COFs nanocomposites for ultrasensitive detection of CRC-derived exosomes. Spherical COFs were functionalized with para-sulfonatocalix [4] arene hydrate (pSC4)-modified gold nanoparticles (AuNPs) and horseradish peroxidase (HRP). The AuNPs, with their excellent conductivity, enhanced the migration rate of charge carriers, thereby improving the biosensor’s response. Meanwhile, pSC4 enabled specific binding to various amino acid residues and their methylated derivatives, facilitating the selective recognition of exosomes. Additionally, the superior biocompatibility and stability of COFs provided an optimal microenvironment for biomolecules. CD63 aptamers were immobilized on the electrode surface to precisely capture exosomes. Tests revealed that the sensor achieved a detection limit as low as 160 particles/μL for CRC-derived exosomes.

In 2022, gastric cancer (GC) accounted for over 968,000 new cases and nearly 660,000 deaths worldwide, making it the fifth most common cancer in terms of incidence and mortality. Due to its frequent diagnosis at advanced stages, gastric cancer is the third leading cause of cancer-related deaths globally [69]. Li et al. [93] introduced an ECL biosensor based on bimetallic MXene-derived quantum dots (Mo_2_TiC_2_ quantum dots) and SnS_2_ nanosheets/lipid bilayers for detecting the gastric cancer biomarker miRNA-27a-3p. The Mo_2_TiC_2_ quantum dots exhibited excellent ECL performance and stability. SnS_2_ nanosheets coated with phospholipid bilayers were designed as a sensing interface. The SnS_2_ nanosheets provided a large surface area and low dielectric constant, effectively reducing luminescence loss and enhancing the luminescence intensity of Mo_2_TiC_2_ (by 2.4 times) while improving the stability of the lipid bilayer. The sensor demonstrated a wide linear range (1 fM to 10 nM) and a low detection limit (1 fM). Additionally, it exhibited high selectivity and reproducibility for miRNA-27a-3p detection.

According to statistics, liver cancer was the sixth most commonly diagnosed cancer globally in 2022 and ranked third in mortality, following lung and colorectal cancers [69]. Alpha-fetoprotein (AFP), a serum protein synthesized by the yolk sac and liver during embryonic development, has been shown to be closely associated with hepatocellular carcinoma (HCC). An AFP concentration exceeding 400 ng/mL is generally considered a reliable diagnostic marker for HCC, making AFP a critical clinical biomarker for liver cancer [94]. Therefore, rapid and sensitive detection of AFP is crucial for HCC diagnosis. Yang et al. [89] developed a ratiometric electrochemical biosensor for AFP detection based on RecJf exonuclease-assisted target recycling and a methylene blue (MB)-DNA-gold nanoparticle (MB-DNA-AuNP) probe combined with a dual-amplification strategy. AFP recognition leads to the dissociation of Fc-CP and triggers RecJf exonuclease-mediated target recycling, significantly amplifying the decrease in Fc signals and the increase in MB signals (Figure 7D). The dual-signal response of Fc and MB provided the sensor with remarkable sensitivity and strong anti-interference capability. The sensor demonstrated a linear detection range spanning seven orders of magnitude and an exceptionally low detection limit of 269.4 ag/mL.

Breast cancer accounts for approximately 30% of cancers in women, making it the second most common cancer globally and the fourth leading cause of cancer-related deaths [69]. Therefore, rapid and accurate detection of breast cancer is crucial. Biomarkers for breast cancer include MUC1, ER, PR, and HER2 [95]. Wang et al. [96] designed an electrochemical sensor for the breast cancer biomarker mucin 1 (MUC1). The sensor utilizes a competitive binding mechanism between complementary DNA-ferrocene (cDNA-Fc)/MXene probes and MUC1 to aptamers (Apt) pre-immobilized on gold nanoparticles (AuNPs) deposited on a glassy carbon electrode (GCE). This enables the quantitative detection of MUC1. The sensor demonstrated a wide linear range (0 pM–10 μM) and an ultralow detection limit of 0.33 pM (S/N = 3).

According to the latest statistics, prostate cancer has become the second most common cancer worldwide and is the most frequently diagnosed cancer among men in 63% of countries [69]. Prostate-specific antigen (PSA), a single-chain polypeptide, is closely associated with prostate inflammation and prostate cancer, making it an essential biomarker for prostate cancer diagnosis. Yang et al. [97] constructed a sandwich-type electrochemiluminescence (ECL) immunobiosensor for detecting PSA. A composite material of poly (indole-6-carboxylic acid) (PICA) with excellent electrochemical activity and flower-like gold nanoparticles (FGNs) with superior conductivity was used to modify the glassy carbon electrode (GCE), providing high conductivity and a large surface area. Additionally, the AuNP/GQDs-PEI-GO composite probe synergistically amplified the ECL signal. The sensor exhibited a wide linear range (0.001 ng/mL to 100 ng/mL) and an ultralow detection limit of 0.44 pg/mL (Table 1).

### 4.2. Infectious Diseases

Infectious diseases are illnesses caused by pathogens including parasites, bacteria, fungi, viruses, or prions that can spread among humans, animals, or between animals and humans [98]. Due to their high transmissibility, long incubation periods, and diverse transmission routes, infectious diseases pose a significant global health burden, threatening human life and health [99]. Over the past decade, outbreaks of infectious diseases such as SARS-CoV-2 (COVID-19), dengue fever, and Ebola virus disease have posed enormous challenges to global public health and the economy. Rapid and highly sensitive detection of pathogens is crucial for the prevention and control of infectious diseases [100]. Compared with traditional diagnostic techniques, electrochemical biosensors offer advantages such as rapid detection, high sensitivity, portability, and low cost, making them highly promising for infectious disease diagnosis.

#### 4.2.1. Coronavirus Disease 2019

Coronavirus disease 2019 (COVID-19) is a viral infectious disease caused by severe acute respiratory syndrome coronavirus 2 (SARS-CoV-2) and is highly contagious and pathogenic. The virus spreads through coughing, sneezing, respiratory droplets, or aerosols entering the human lungs [101]. The pathogenesis of SARS-CoV-2 infection involves varying degrees of respiratory system failure. After attaching to the surface of respiratory epithelial cells, the virus replicates and gradually invades the lower respiratory tract, ultimately infecting alveolar epithelial cells [102]. Therefore, designing a sensor capable of rapid, accurate, and highly specific detection of SARS-CoV-2 is critical. Li et al. [103] presented an ultrasensitive electrochemical biosensor for SARS-CoV-2 RNA detection based on a capture probe–target RNA-labeling probe sandwich structure. The capture probe (CP) was immobilized on the surface of Au@Fe_3_O_4_ nanoparticles. The labeling probe (LP), combined with crown ether-functionalized graphene oxide, formed a Au@SCX8-TB-RGO-LP bioconjugate. p-Sulfonatocalix [8] crown ether (SCX8) enriched the signal molecule toluidine blue (TB), further enhancing the electrochemical signal, while reduced graphene oxide (RGO) provided high conductivity and a large surface area, improving electron transfer efficiency. The sensor demonstrated an ultralow detection limit of 200 copies/mL for SARS-CoV-2 in clinical samples. Moreover, the positive detection rates for confirmed and recovered patients reached 85.5% and 46.2%, respectively, outperforming RT-qPCR. Additionally, the sensor can directly detect clinical samples without RNA amplification, offering a system for rapid, on-demand SARS-CoV-2 detection.

**Figure 8:**
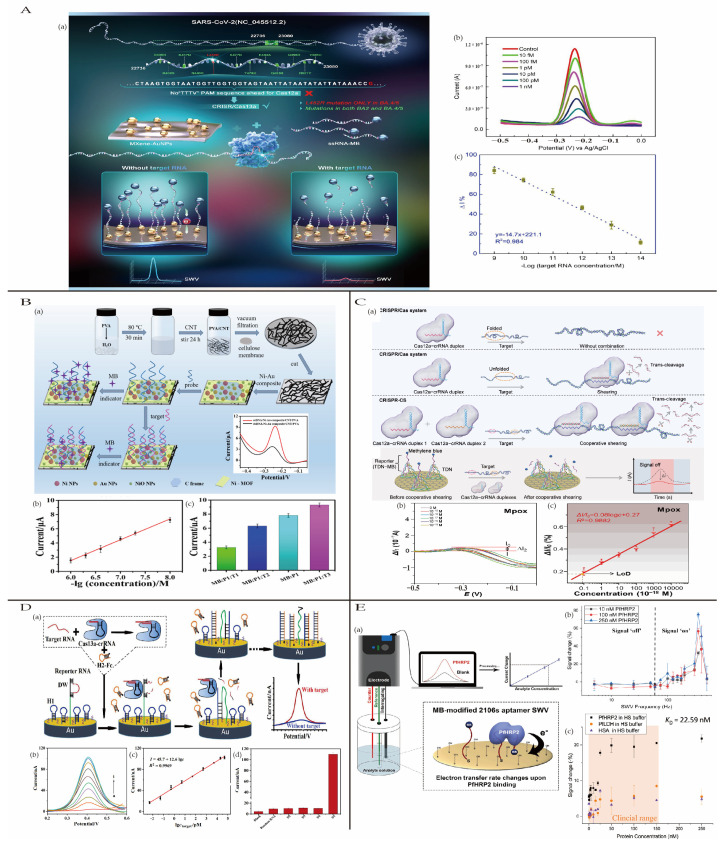
(**A**) (**a**) The following schematic illustration details the process of nucleic acid detection for SARS-CoV-2, as outlined in the MoECS, utilizing the CRISPR-Cas13a system. (**b**) The SWV curves demonstrate target RNA concentrations ranging from 1nM to 10 fM. The control condition involves the replacement of target RNA with pure H_2_O. (**c**) The equation demonstrates a linear relationship between the change in current (Δ I%) and the logarithm of the target RNA concentration; reproduced with permission from [106]. Copyright 2023, Springer Nature. (**B**) (**a**) The schematic illustrates the manufacturing process for the flexible Ni–Au composite/CNT/PVA film electrode and the detection of the target DNA; (**b**) DNA biosensor calibration curves for increasing concentrations of target DNA; (**c**) the selective targeting of DNA sequences with flexible paper-based electrodes; reproduced with permission from [109]. Copyright 2021, Elsevier. (**C**) Working principle of the CRISPR-CS system. (**a**) Reaction principle of the conventional CRISPR/Cas12a shearing system and CRISPR-CS system; (**b**) schematic of the shearing and testing process; (**c**) Lod of CRISPR-CS; reproduced with permission from [113]. Copyright 2024, John Wiley and Sons. (**D**) The CRISPR-Cas system concentration response against Mpox. (**a**) A schematic representation of electrochemical biosensor production and the detection of DENV RNA using CRISPR/Cas13a-assisted CHA. (**b**) ACV responses of biosensor to different concentrate DENV-1 under optimal conditions. (**c**) Plot of peak current vs. logarithm of DENV-1 concentration. (**d**) Specificity of biosensor against 50 nM Random RNA, DENV, DENV-3, and DENV-4, and 5 nM DENV-1. Reproduced with permission from [114]. Copyright 2021, Elsevier. (**E**) (**a**) The schematic workflow of the E-AB biosensor platform; (**b**) the MB peak signals of the 2106 s E-AB biosensor were examined at concentrations of 10, 100, and 250 nM PfHRP-2 in PBS; (**c**) the HS buffer background concentration response curve. Reproduced with permission from [115]. Copyright 2021, Elsevier.

Omicron, a variant of SARS-CoV-2, exhibits stronger transmissibility and immune evasion capabilities compared to the original SARS-CoV-2 [104]. Studies have identified the L452R mutation as a crucial marker for detecting the SARS-CoV-2 Omicron BA variant [105]. Chen et al. [106] utilized a CRISPR/Cas13a system with trans-cleavage activity for highly specific detection of the SARS-CoV-2 Omicron BA.5 variant. The working electrode (AuE) was functionalized with an MXene-AuNP composite, enhancing its electrochemical surface area and conductivity. The sensor demonstrated a detection limit of 1 fM, with a good linear relationship in the range of 1 nM to 10 fM. Additionally, the sensor was selectively sensitive to the L452R mutation present in the Omicron BA.5 variant (Figure 8A). This biosensor also exhibited rapid detection capability (within one hour) and long-term stability. Validation using clinical samples showed results consistent with qPCR, confirming its practical utility.

#### 4.2.2. Acquired Immune Deficiency Syndrome

Human immunodeficiency virus (HIV) compromises the immune system, causing Acquired Immune Deficiency Syndrome (AIDS), which is infectious. According to the latest statistics, as of 2021, the global number of people living with HIV reached 40 million (38–42.4 million), with an estimated 1.43 million (1.29–1.59 million) new HIV infections and 615,000 (567,000–680,000) AIDS-related deaths predicted for 2025. Research has shown that early screening and diagnosis are critical for the treatment of AIDS [107]. Zhou et al. [108] developed a dual-mode electrochemiluminescence (ECL) and photoelectrochemical (PEC) biosensor for HIV detection. The sensor utilized 3D CdSe quantum dots (QDs) as efficient photosensitive materials and a DNA nanonetwork-functionalized SnO_2_ nanoflower as the PEC substrate. The SnO_2_ and CdSe QDs coupled to form a cascade band, promoting effective transfer of photogenerated carriers. The biped DNA walker cascade amplification strategy was employed, generating a large number of DNA strands and attaching abundant signal probes and quantum dots to the electrode, significantly enhancing the sensor’s sensitivity. The sensor achieved a linear detection range of 0.5 μM to 5 fM and a detection limit of 1.38 fM, with excellent specificity, stability, and potential for practical applications. Additionally, Shu et al. [109] used a nickel metal–organic framework, (Ni-MOF)/gold nanoparticles/carbon nanotubes/polyvinyl alcohol (Ni–Au composite/CNT/PVA), to construct a flexible paper-based electrode for HIV DNA detection (Figure 8B). CNT/PVA films were vacuum-filter deposited onto cellulose membranes, followed by drop-casting Ni–Au composite materials onto the CNT/PVA film electrodes to form a flexible composite membrane (CCP). The CNT/PVA film near the cellulose membrane provided good conductivity and biocompatibility, while the Ni-MOF/gold nanoparticle composite with a conjugated π–electron system and large surface area allowed for higher probe DNA loading, enhancing sensitivity. The sensor demonstrated a wide linear detection range (10 nM–1 μM) and a low detection limit of 0.13 nM, with high specificity for fully complementary target HIV DNA, as well as good reproducibility and long-term stability.

#### 4.2.3. Monkeypox

Monkeypox is a zoonotic viral infection caused by the monkeypox virus (Mpox), a member of the orthopoxvirus genus related to the smallpox virus. It was first identified in humans in 1970 [110]. The virus primarily spreads through respiratory droplets, direct contact, and contact with contaminated objects. Since 2022, monkeypox outbreaks have occurred in multiple countries, prompting the World Health Organization (WHO) to declare it a global public health emergency [111]. Currently, there is no effective treatment for monkeypox, making early diagnosis and virus control crucial [112]. Wei et al. [113] developed a CRISPR/Cas cooperative shearing (CRISPR-CS) system to address challenges related to target DNA folding during nucleic acid detection. The CRISPR-CS system utilizes two Cas12a-crRNA duplexes that efficiently bind to two distinct sites on the target DNA, enhancing binding and cleavage efficiency. Using monkeypox virus nucleic acid as the target analyte, the feasibility of the system was validated. The CRISPR-CS-based electrochemical biosensor demonstrated an ultralow detection limit of 9.5 × 10^−20^ M, which is 2 to 9 orders of magnitude lower than other electrochemical detection methods (Figure 8C). Additionally, this system was also shown to detect nucleic acids of human papillomavirus (HPV) and amyotrophic lateral sclerosis (ALS), indicating its potential as an ideal platform for nucleic acid detection.

#### 4.2.4. Dengue Fever

Dengue fever, caused by the dengue virus (DENV), is an acute infectious disease, transmitted through mosquito bites. Approximately 400 million people worldwide are affected by dengue fever annually. Without timely treatment, patients may develop severe complications or even face fatal outcomes [116]. Therefore, rapid and sensitive early diagnosis of dengue fever is critical. Ju et al. [114] developed a sensor for sensitive detection of DENV based on CRISPR/Cas13a-assisted catalytic hairpin assembly (CHA) and a DNA walker (DW). In the presence of DENV, the Cas13a protein is activated to cleave reporter RNA, releasing the walker. The walker binds to hairpin 1 (H1), triggering a strand displacement reaction and further amplifying the signal (Figure 8D). The sensor demonstrated a linear detection range of 5 fM to 50 nM and an ultralow detection limit of 0.78 fM.

#### 4.2.5. Malaria

Malaria, caused by Plasmodium parasites, is an acute infectious disease and it is primarily transmitted through mosquito bites, blood transfusion, or the use of contaminated needles. In 2022, malaria resulted in over 600,000 deaths. Rapid and accurate early diagnosis of malaria is crucial for preventing severe disease and potential fatalities [117]. Key biomarkers for malaria detection include parasite lactate dehydrogenase (pLDH), Plasmodium falciparum histidine-rich protein 2 (PfHRP-2), the biocrystal hemozoin, aldolase, and glutamate dehydrogenase (GDH) [118]. Lo et al. [115] developed a bioelectrochemical sensor based on a 2106s aptamer (E-AB) for the rapid detection of the malaria biomarker PfHRP-2. The aptamer, selected through SELEX (Systematic Evolution of Ligands by Exponential Enrichment), exhibited high binding affinity to PfHRP-2. The sensor demonstrated a limit of detection (LOD) of 3.73 nM for PfHRP-2 in complex serum backgrounds and showed excellent stability, enabling multiple reuse cycles (Figure 8E). Additionally, the sensor achieved a fast detection time (5–6 min), significantly outperforming traditional detection methods and other electrochemical biosensors for PfHRP-2 (Table 2).

### 4.3. Inflammatory Diseases

Inflammation is a physiological defense response of the body to harmful external stimuli, such as infections, injuries, toxins, or physical trauma. Its purpose is to protect the body by eliminating pathogenic factors, repairing damaged tissues, and restoring normal physiological functions. While inflammation is a necessary physiological reaction, in some cases, it can lead to severe tissue necrosis or even death [98]. Therefore, the early, rapid, and accurate diagnosis of inflammation is critical for its prevention and treatment.

#### 4.3.1. Sepsis

**Figure 9:**
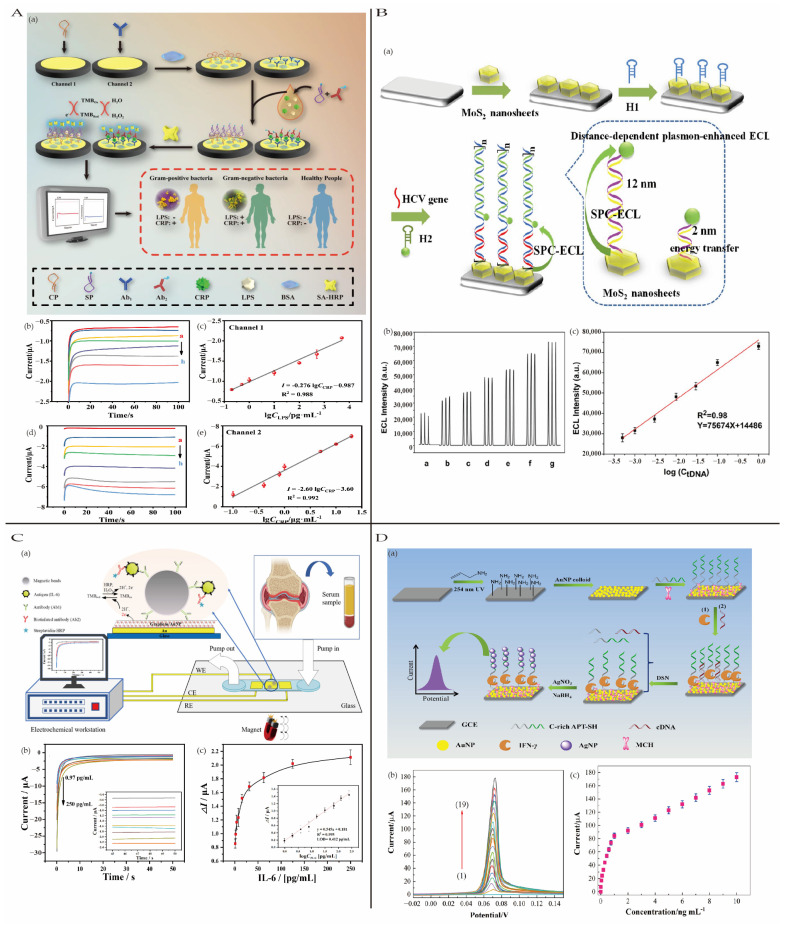
(**A**) (**a**) A diagram of the fabrication step of a dual-channel electrochemical biosensor for the synchronous detection of LPS and CRP. The i-t curves (**b**) and corresponding current-concentration linear fitting curve (**c**) of the electrochemical biosensor for LPS at a range of concentrations; the i-t curves (**d**) and corresponding current-concentration linear fitting curve (**e**) of the electrochemical biosensor for CRP at a range of concentrations. Reproduced with permission from [122]. Copyright 2024, Elsevier. (**B**) (**a**) The HCR-based detection method and distance-dependent plasmon-enhanced ECL; (**b**) the correlation between HCV gene concentration and ECL intensity; (**c**) the linear relationship with the logarithm of the concentration of the HCV gene in the range of 0.5 pM–1 Nm; reproduced with permission from [127]. Copyright 2020, Elsevier. (**C**) (**a**) Scheme illustrating the design and sensing of our electrochemical magnetoimmunosensor for IL-6 detection; (**b**) the amperometric responses of TMB_ox_ in PBS at different IL-6 concentrations (pg/mL) from top to bottom; (**c**) the electrochemical magnetoimmunosensor calibrated to target IL-6; reproduced with permission from [129]. Copyright 2022, Elsevier. (**D**) (**a**) Schematic representation of the aptasensing strategies for detection of IFN-*γ*; (**b**) LSV curves of the electrochemical assay for the detection of IFN-*γ* with different concentrations; (**c**) relationship between the peak current and IFN-γ concentration; reproduced with permission from [130]. Copyright 2022, Elsevier.

Sepsis is an uncontrolled systemic inflammatory response primarily triggered by pathogens such as fungi, microorganisms, viruses, and parasites. This response often causes severe damage to the body’s tissues and organs, potentially leading to death [119]. It is estimated that 5.3 million people die from sepsis annually worldwide [120]. Additionally, neonatal sepsis remains a significant concern, threatening the lives of 5 million children each year [121]. Therefore, early detection of sepsis is crucial. Liu et al. [122] developed an electrochemical biosensor based on a dual-channel strategy for the simultaneous detection of lipopolysaccharides (LPSs) and C-reactive protein (CRP). Independent detection of LPS and CRP was achieved using a sandwich strategy with suitable specific aptamers, establishing a sensitive and rapid diagnostic method for sepsis and Gram staining-based classification to better guide treatment (Figure 9A). The biosensor exhibited linear detection ranges of 0.5–1000 pg/mL with a detection limit of 0.343 pg/mL and 0.1–20 μg/mL with a detection limit of 0.05 μg/mL for LPS and CRP.

#### 4.3.2. Viral Hepatitis

Viral hepatitis, caused by viral infections, is an inflammation of the liver, and it is common worldwide. It damages liver cells and leads to abnormal liver function. Hepatitis B and C viruses are major causes of primary hepatocellular carcinoma cases. According to statistics, diseases related to hepatitis B and C viruses caused 1.1 million deaths in 2020 [123]. Therefore, rapid and accurate early diagnosis of viral hepatitis is imperative.

Hepatitis B virus (HBV) is a non-cytopathic, hepatotropic virus that can lead to persistent infections, ultimately causing complications such as liver cirrhosis and hepatocellular carcinoma [124]. Yuan et al. [125] utilized boron and nitrogen co-doped carbon dots (BN-CDs) to create a novel electrochemiluminescence (ECL) emitter with low excitation potential and high ECL efficiency. By integrating this novel ECL ternary system with an exonuclease III (Exo III)-induced target DNA amplification strategy, they achieved ultrasensitive detection of HBV DNA. In this system, S_2_O_8_^2−^ served as the core reactant. Platinum nanoflowers (Pt NFs) and boron radicals (B•) generated from B doping in BN-CDs synergistically accelerated the reduction in S_2_O_8_^2−^, producing abundant SO_4_•⁻ radicals, which significantly enhanced the ECL efficiency of BN-CDs. The ECL biosensor demonstrated a linear detection range of 100 aM - 1 nM for HBV DNA, with a detection limit of 18.08 aM, offering a promising and highly efficient ECL emitter for sensitive diagnostics.

Hepatitis C virus (HCV) is an RNA virus and one of the leading causes of chronic liver disease worldwide. HCV infection can result in liver fibrosis, cirrhosis, and even hepatocellular carcinoma (HCC). Early diagnosis of HCV infection is crucial for timely and effective treatment [126]. Liu et al. [127] constructed a surface plasmon coupling (SPC)-enhanced ECL biosensor by optimizing the distance between non-metallic plasmonic MoS_2_ nanosheets and sulfur-doped boron nitride quantum dots (S-BN QDs). This optimization significantly enhanced the electrochemiluminescence (ECL) signal of S-BN QDs, with notable ECL enhancement observed at distances between 12.5 nm and 20 nm (Figure 9B). Additionally, a hybridization chain reaction (HCR) was employed for the sensitive detection of the target HCV gene. The ECL biosensor achieved a linear detection range of 0.5 pM to 1 nM for the HCV gene, with a limit of detection (LOD, S/N = 3) of 0.17 pM.

#### 4.3.3. Other Inflammatory Diseases

Rheumatoid arthritis (RA) is an autoimmune disease which affects approximately 1% of the adult population, primarily characterized by chronic inflammation of the joints, resulting in joint damage and disability in severe cases. Studies have shown that early diagnosis and treatment of RA can prevent or significantly slow the progression of joint damage in up to 90% of patients, thereby reducing the risk of irreversible disability [128]. Elevated levels of the cytokine Interleukin-6 (IL-6) in RA patients make it a valuable biomarker for the early diagnosis of RA. Zhang et al. [129] presented a microfluidic-based electrochemical magnetic immunosensor (EC-MIS) for the detection of IL-6, using a AuNPs/graphene hybrid to modify the working electrode to improve the conductivity and sensitivity of the sensor.

Additionally, capture antibodies (cAb) were conjugated with magnetic beads to improve the enrichment of the analyte IL-6. The sensor exhibited a linear detection range of 0.97–250 pg/mL, and LOD is 0.42 pg/mL (Figure 9C). Furthermore, it demonstrated feasibility and effectiveness in practical applications.

Acute myocarditis is an acute injury to myocardial cells induced by viruses, bacteria, immune-mediated diseases, or drugs, leading to severe inflammation that can progress to symptomatic heart failure and even death [131]. Circulating miR-721 has been identified as a novel diagnostic biomarker for acute myocarditis and can be used for early diagnosis and treatment guidance. Wang et al. [132] developed an ultrasensitive non-enzymatic triple amplification electrochemical biosensor for miR-721 detection, integrating a molecular beacon-based catalytic hairpin assembly (mCHA) circuit with MOF@Au@G-triplex/hemin nanozymes. By introducing a signal transduction probe (HpC) into the catalytic hairpin assembly (CHA), a molecular beacon-based CHA (mCHA) circuit was designed, enhancing the flexibility of the CHA amplification circuit. The sensing film, composed of Au nanoparticles and functionalized g-C_3_N_4_ nanosheets (NSs), improved conductivity, while nitro-functionalized iron-based tetrahedral DNA nanostructures (TDNs) optimized probe orientation and electron transfer. The MOF@Au and G-triplex/hemin-based nanozymes catalyzed the oxidation of TMB, significantly amplifying the signal. Additionally, the use of magnetic bead separation technology increased reaction efficiency. The biosensor exhibited an ultralow detection limit of 0.25 fM for miR-721 and demonstrated excellent selectivity, good reproducibility (relative standard deviation of 3.46% across five parallel experiments), and strong stability.

#### 4.3.4. Inflammatory Biomarkers

C-reactive protein (CRP) is a spherical pentameric protein primarily synthesized in the liver and serves as a key regulator during inflammation. Elevated levels of CRP in the blood are a clear indicator of inflammation following tissue injury, making it a nonspecific diagnostic biomarker for rheumatoid arthritis and other inflammatory conditions [133]. Shui et al. [134] developed a high-performance aptasensor for CRP detection based on AuNPs@C-ZIF67 composites. The rhombic dodecahedral carbonized C-ZIF67 composite loaded with gold nanoparticles served as the sensing platform, offering a high surface area and excellent electron transfer performance. Horseradish peroxidase-labeled CRP antibodies (HRP-AbCRP) were introduced as detection probes to ensure the specificity of the sensor. An enzyme-catalyzed signal amplification mechanism further enhanced the sensitivity and signal intensity of the electrochemical biosensor. After optimization, the sensor exhibited a linear detection range of 10 pg/mL to 10 μg/mL, with a detection limit of 0.44 pg/mL (S/N = 3). Additionally, it enabled rapid detection of human plasma samples within 5 min.

Interferon-γ (IFN-γ) is a critical multifunctional cytokine secreted by natural killer cells, T cells, and mucosal epithelial cells. It is an important early biomarker for inflammation and various diseases [135]. Therefore, the rapid, simple, and highly sensitive detection of IFN-γ is essential for the prevention and treatment of inflammatory conditions. Xu et al. [130] developed an electrochemical biosensor for IFN-γ detection based on a thiolated-modified DNA aptamer with a C-rich sequence (C-rich Apt-SH). Upon recognition of IFN-γ, the aptamer triggers the release of C-rich fragments through double-strand-specific nuclease (DSN) activity. These fragments act as templates for the in situ formation of silver nanoclusters (AgNCs) on the electrode surface in the presence of AgNO_3_ and NaBH_4_, enabling the electrochemical detection of IFN-γ (Figure 9D). Additionally, gold nanoparticles (AuNPs) modified on the electrode provided high conductivity and a large surface area, further enhancing the sensor’s performance. The biosensor exhibited a detection limit of 1.7 pg/mL for IFN-γ, with excellent specificity and stability, demonstrating potential for detection in complex environments.

Interleukin-6 (IL-6) is both a pro-inflammatory and anti-inflammatory cytokine, serving as an inducer of acute-phase responses and a key regulator of specific cellular and humoral immune responses. It plays a central role in inflammatory reactions, immune regulation, and tissue repair [136]. Abnormal levels of IL-6 are associated with various diseases, including inflammation, making its highly sensitive detection critically important [137]. Chen et al. [138] proposed an ultrasensitive immunosensor based on the Ab-SPA-Cys-AuNPs-THI-CMWCNTs nanocomposite for IL-6 detection. The sensor’s substrate, AuNPs-THI-CMWCNTs nanohybrids, provides excellent conductivity, biocompatibility, and antibody immobilization efficiency. Staphylococcal protein A (SPA) was used to directionally immobilize antibodies, effectively reducing steric hindrance and enhancing the binding capacity for target analytes. The sensor demonstrated a detection limit of 2.87 pg/mL for IL-6, with a linear detection range of 0.01 ng/mL to 800 ng/mL. Furthermore, it successfully quantified IL-6 concentrations in serum samples from myocardial infarction rats and various tissue lysates, extending its potential applications. Liu et al. [139] designed a label-free electrochemical immunosensor based on nanoporous carbon composites (NMCs) and gold nanoparticles (AuNPs) in NMC@AuNP composites for the rapid and ultrasensitive detection of Interleukin-6 (IL-6), with a linear dynamic range of the sensor from 0.5 to 1200 pg/mL and a detection limit (LOD) as low as 0.14 pg/Ml (Table 3).

### 4.4. Neurodegenerative Diseases

Neurodegenerative diseases (NDDs) are a heterogeneous group of neurological disorders characterized by the gradual degeneration or loss of neurons in the central nervous system (CNS) or peripheral nervous system (PNS). The structural and functional collapse of neural networks is the result of this degeneration, ultimately resulting in the complete loss of cognitive, motor, sensory, behavioral, and emotional functions [140]. Common neurodegenerative diseases include Alzheimer’s disease (AD), Parkinson’s disease (PD), and amyotrophic lateral sclerosis (ALS). Early and accurate diagnosis of neurodegenerative diseases is crucial for their classification, diagnosis, and subsequent treatment.

#### 4.4.1. Alzheimer’s Disease

**Figure 10:**
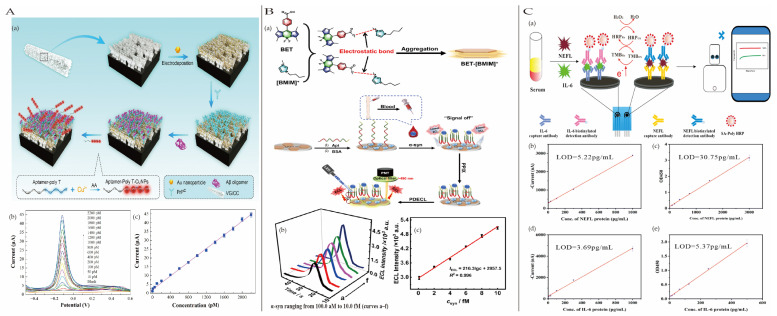
(**A**) (**a**) A schematic representation of the electrochemical sensor for the detection of Aβ oligomers; (**b**) electrochemical assay DPV curves corresponding to different concentrations of Aβ oligomers; (**c**) the corresponding Aβ oligomer detection calibration curve; reproduced with permission from [143]. Copyright 2021, Elsevier. (**B**) (**a**) Synthesis of the BET-[BMIm] and stepwise fabrication of the “Signal off” Model and PDECL Device for Ultrasensitive Analysis of α-Syn; (**b**) the ECL responses of the as-built PDECL biosensor for the determination of α-syn from 100.0 aM to 10.0 fM.; (**c**) the calibration curve; reproduced with permission from [148]. Copyright 2024, American Chemical Society. (**C**) (**a**) A scheme of the smartphone-based electrochemical biosensor for the direct detection of the serum biomarkers NEFL and IL-6; electrochemical biosensor dose–response curves for NEFL detection (**b**) and ELISA (**c**). Based on the electrochemical biosensor, the dose–response curves for IL-6 detection (**d**) and ELISA (**e**). Reproduced with permission from [149]. Copyright 2024, American Chemical Society.

Alzheimer’s disease (AD) is one of the most common neurodegenerative diseases and a leading cause of dementia in the elderly, representing about 60–80% of all dementia cases. It affects an estimated 50 million people worldwide, with the number expected to triple by 2050 [141]. AD has a long latency period, during which brain changes occur 20 years or more before symptoms appear, often unnoticed by affected individuals. Eventually, it leads to significant symptoms such as memory loss, language impairment, and even death [142]. Although the exact cause of AD remains unclear, it can be rapidly and accurately diagnosed by detecting biomarkers in cerebrospinal fluid (CSF) such as total tau protein (T-tau), amyloid-beta (Aβ42), and phosphorylated tau protein (P-tau) [141]. Xu et al. [143] developed a highly sensitive biosensor for detecting Amyloid-beta (Aβ) oligomers, a biomarker of AD, using differential pulse voltammetry (DPV). The biosensor utilized a 3D vertically grown graphene (VG) electrode substrate uniformly deposited on carbon cloth (CC) and further enhanced with electrodeposited gold nanoparticles, providing a high surface area and excellent electrical conductivity. Aβ oligomer receptors were immobilized on a poly-thymine (poly T)-modified DNA aptamer, enhancing the specificity and sensitivity of the sensor (Figure 10A). The biosensor achieved an ultralow detection limit of 3.5 pM for Aβ oligomers and showed no significant differences when compared to ELISA kits in testing human serum samples.

Zhang et al. [144] designed a single electrochemical array based on microcolumns to simultaneously detect multiple biomarkers. The array anchors liquid droplets in open-channel microreactors, enabling multiplexed detection. The working electrode of each polydimethylsiloxane (PDMS) microcolumn sensor was modified with gold nanomaterials via electrodeposition. Specific antibodies and DNA probes were further immobilized to enable the sensitive and simultaneous detection of four Alzheimer’s disease biomarkers: Tau, ApoE4, amyloid-beta (Aβ), and miRNA-101.

#### 4.4.2. Parkinson’s Disease

Parkinson’s disease (PD) ranks as the second most prevalent neurodegenerative disorder and a leading cause of neurological disability, characterized by motor impairments such as bradykinesia, muscle rigidity, resting tremor, and postural and gait instability [145]. According to the latest statistics, the prevalence and mortality rates of PD are rapidly increasing worldwide, with over 6 million individuals affected globally [146]. The primary pathological features of PD include neurodegeneration in the substantia nigra pars compacta and the accumulation of synuclein aggregates. Among these, α-synuclein oligomers are considered one of the most promising candidate biomarkers. Liao et al. [147] prepared an electrochemical biosensor based on a DNAzyme-driven bipedal DNA walker for the detection of α-synuclein oligomers. This biosensor utilizes the bipedal DNA walker and a DNAzyme-powered cleavage process to convert the presence of target molecules into exponentially amplified electrochemical signals, significantly enhancing detection sensitivity and signal strength. The sensor exhibited a linear detection range for α-synuclein oligomers from 1 fg/mL to 10 pg/mL, with a detection limit of 0.57 fg/mL. Additionally, due to the high affinity of antibodies for α-synuclein oligomers, the sensor demonstrated excellent selectivity and anti-interference capabilities, highlighting its significant potential for practical applications in biological environments.

Additionally, Song et al. [148] developed a highly sensitive electrochemical biosensor for the detection of the key Parkinson’s disease biomarker α-synuclein (α-syn), utilizing the photodynamic-assisted electrochemiluminescence (PDECL) enhancement principle. The biosensor employs an advanced emitter prepared by electrostatically linking 2,6-dimethyl-8-(3-carboxyphenyl)-4,4′-difluoroboradiazene (BET) with 1-butyl-3-methylimidazole tetrafluoroborate ([BMIm][BF4]). Using the photosensitizer protoporphyrin IX (PPIX), the system generates singlet oxygen (^1^O_2_) under light irradiation, significantly enhancing the electrochemiluminescence (ECL) signal of the emitter. The aptamer targeting α-syn enables specific recognition, achieving high sensitivity in α-syn detection. The sensor demonstrated a detection limit as low as 63 aM, with a linear range of 100.0 aM to 10.0 fM (Figure 10B). It successfully differentiated serum samples from Parkinson’s disease patients and healthy controls, achieving a diagnostic accuracy (AUC) of 0.994.

#### 4.4.3. Other Neurodegenerative Disease Biomarkers

Neurofilament light chain (NEFL) is a neuron-specific structural protein, and its serum levels are correlated with the severity of inflammation in neurodegenerative diseases, making it a potential biomarker for these conditions. Song et al. [149]. reported a novel portable electrochemical biosensor designed for highly sensitive and specific detection of NEFL and IL-6 in serum. The biosensor utilizes a double-antibody sandwich structure formed by capture and detection antibodies on the sensor surface, coupled with enzyme-catalyzed signal amplification to enhance electrochemical signal detection. The sensor achieved detection limits of 5.22 pg/mL for NEFL and 3.69 pg/mL for IL-6. In blood sample testing, the detection rates for both biomarkers were comparable to or even superior to those of commercial ELISA kits (Figure 10C). Additionally, the biosensor can be connected to a smartphone, enabling real-time monitoring and early prevention of neurological diseases.

Dopamine (DA) is a critical neurotransmitter and neuromodulator that plays a dual role in neurodegenerative diseases, serving as both a key participant in pathological mechanisms and a central therapeutic target [150]. Monitoring DA levels in vivo is essential for the diagnosis and prevention of neurodegenerative disorders. Wang et al. [151] developed a highly sensitive sensor for DA detection based on electrochemical surface plasmon resonance (EC-SPR) technology. The sensor was developed by electropolymerising DA in the presence of 6-mercaptohexanoic acid (MHA) to polydopamine (PDA), which alters the plasmonic resonance (SPR) signal intensity on the gold film surface. Remarkably, the sensor remains unaffected by high concentrations of interfering substances, such as uric acid and ascorbic acid. The sensor achieved a detection limit as low as 1.4 pM, significantly surpassing traditional electrochemical detection methods, and demonstrated high selectivity along with a wide linear range (0.01–1000 nM) (Table 4).

## 5. Summary and Outlook

The simplicity, low cost, and portability of electrochemical biosensors make them an attractive method for disease diagnosis. However, they still have problems such as limited stability, reproducibility, and durability, low detection accuracy in complex samples, and difficulties in complex data analysis and processing, making it difficult for most research to achieve commercial development. Secondly, the regulation of electrochemical biosensors in clinical applications is highly stringent. In many regions, wearable and implantable biosensors for medical purposes must undergo rigorous testing and approval processes to ensure long-term wear does not pose risks to human health. Although these regulations are essential for consumer safety, prolonged approval periods may delay market entry, reducing the device’s relevance and competitiveness. Moreover, significant variations in safety standards across different regions further increase uncertainty in the global market. In addition, with the growing adoption of wearable devices and telemedicine, health data are now frequently transmitted to mobile devices or cloud platforms in real time. The privacy and security of consumers’ health data represent another major challenge in the commercialization of electrochemical biosensors. To protect personal health information, strict data encryption and privacy protection protocols must be implemented during data transmission and storage. Developing a standardized data security framework is crucial for the sustainable growth of industry.

Although the work of these Chinese groups cited in this review has broadened the application prospects of electrochemical biosensors to a certain extent, current research still has certain limitations. First, the current research on electrochemical biosensors aims to improve the sensitivity and selectivity of sensors, but few of these works can directly detect samples without pretreatment, which greatly limits the practical application and commercial development of electrochemical biosensors. The high concentration and variety of nonspecific contamination in complex samples can easily lead to inaccurate results. Although many strategies have been explored to solve this problem, such as hydrogels, selective ion membranes, and surface hydrophilic/hydrophobic properties adjustment, these methods may hinder the contact between the target molecules and the sensing layer, resulting in decreased sensitivity or insufficient long-term stability. More stable and long-term methods are needed to improve the anti-fouling performance of sensors. Chinese research groups have explored this aspect. For example, Ding et al. [152] used a ratio strategy to effectively reduce background interference and designed a multifunctional peptide with hydrophilicity and neutrality, which gave the sensor good anti-fouling ability. In addition, although electrochemical biosensors are less expensive than traditional detection instruments, for commercial applications, the regenerative detection of sensors can achieve higher cost-effectiveness and more frequent instant diagnosis. Each time it is used, the interfering substances in the sample will be non-specifically adsorbed on the electrode surface, and long-term accumulation will cause the electrode to respond to the target molecule. On the other hand, the electrochemical reaction is carried out on the electrode for a long time, and the electrode surface may be physically or chemically damaged, which will also affect the repeatability of the sensor. In this regard, the Chinese research group Liu et al. [153] proposed a “biphasic replacement” E-AB (BRE-AB) sensing platform for high-sensitivity detection of picomolar biomacromolecules. This strategy places the biomacromolecule capture reaction in a homogeneous solution phase and replaces it with a small-diameter single-stranded DNA to attach to the interface, achieving low cost, easy regeneration, and reusability of the sensor. In addition, many diseases are not caused by a single factor and may involve many biological pathways. And a single disease marker is easily affected by other physiological processes. The simultaneous detection of multiple biomarkers is crucial for accurately determining disease subtypes and the severity of the disease. The Chinese research group Xie et al. [154] developed a vertical graphene-based multi-parameter sensor array (VG-MSA) for peritoneal dialysis monitoring. The sensor integrates multiple detection modules and can detect a variety of metabolites and ions, providing a valuable tool for monitoring dialysis quality and adjusting treatment plans.

Electrochemical biosensors are currently facing severe challenges, but their advantages in disease diagnosis make them have great potential in human health monitoring, early disease diagnosis and clinical application. Although my country’s electrochemical sensor technology started late, my country’s high-tech technologies, such as micro-nano manufacturing, artificial intelligence, and nanomedicine, have developed rapidly, providing new opportunities and challenges for electrochemical biosensors. Traditional electrochemical biosensors rely on a single type of signal for measurement and analysis, which is easily interfered with by external factors. Machine learning algorithms classify, denoise, and extract feature data for complex multi-dimensional data, which can better interpret the relationship between data. In addition, we can also use model training and other methods to enable it to process input data in real time and evaluate the data. The integration of machine learning algorithms into electrochemical biosensors can significantly improve the efficiency and accuracy of disease diagnosis, especially in complex pathological environments. Flexible wearable devices are a prerequisite for accurate human health monitoring, and their development relies on innovative optimization of sensor materials and structural design. For example, the use of advanced materials such as conductive gels and liquid metal electrodes can significantly improve the bending resistance, stretchability, biocompatibility, and wearing comfort of sensors. Secondly, the lack of standardization remains a major obstacle to commercial applications. This includes the lack of unified performance evaluation indicators, such as the quantitative relationship between the bending frequency and signal drift of flexible sensors and the sensitivity threshold to temperature and humidity fluctuations. In addition, differences in data formats and transmission protocols between different manufacturers hinder the integration of medical platforms. A common communication protocol is urgently needed to enhance industry-wide interoperability.

## Figures and Tables

**Table 1:** Summary of electrochemical sensors used for cancer detection in this chapter.

Cancer	Biomarker	Materials	Detection Methods	LOD	Range	Ref.
APL	PML/RARα fusion gene	CDs/GO/GCE	DPV	83 pM	2.5 × 10^−10^–2.25 × 10^−9^ M	[74]
CML	BCR/ABL fusion gene	Ti_3_C_2_T_x_ MXene	DPV	0.05 fM	0.2 fM–20 nM	[76]
CML	BCR/ABL^p210^ transcript	Enzyme-linked DNA-coating MBs	CA	1 aM	1.0 × 10^−18^–5.0 × 10^−14^ M 1.0 × 10^−15^–1.0 × 10^−12^ M	[77]
NSCLC	miRNA	G-quadruplex/dsDNA	LSV	5.68 fM	20 fM to 5 nM	[81]
NSCLC	CYFRA21-1	CD-MOF@Ru(bpy)_3_^2+^	ECL	0.006 ng/mL	0.1–50 ng/mL	[82]
OC	HE4	TiMOF-KB@AuNPs	DPV	0.02 ng/mL	0.1–80 ng/mL	[87]
OC	CA125	AuNFs@MoS_2_/CA125 aptamer/MCH	DPV	0.0001 U/mL	0.0001–35 U/mL 35–500 U/mL	[88]
CRC	Exosomes	HRP-pSC_4_-AuNPs@COFs	CA	160 particles/μL	5 × 10^2^ to 10^7^ particles/μL	[92]
GC	miRNA-27a-3p	Mo_2_TiC_2_ QDs	ECL	1 fM	1 fM–10 nM	[93]
Liver cancer	AFP	MB-DNA-AuNP	ACV	269.4 ag/mL	10 fg/mL–100 ng/mL	[89]
Breast cancer	MUC1	cDNA-ferrocene/MXene	SWV	0.33 pM	1.0 pM–10 μM	[96]
Prostate cancer	PSA	AuNP/GQDs-PEI-GO	ECL	0.44 pg/mL	0.001 ng/mL–100 ng/mL	[97]

**Table 2:** Summary of electrochemical sensors used for infectious disease detection in this chapter.

Infectious Diseases	Biomarker	Materials	Detection Methods	LOD	Range	Ref.
COVID-19	SARS-CoV-2 RNA	SCX8-RGO	DPV	200 copies/mL	10^−17^–10^−12^ M	[103]
Omicron	crRNA	AuE-MXene-AuNPs	SWV	1 fM	1 nM–10 fM	[106]
AIDS	HIV DNA	3D CdSe QDs-DNA/SnO_2_ nanolowers	ECL/PEC	1.38 fM	0.5 μM–5 fM	[108]
AIDS	HIV DNA	Ni-MOF/AuNPs/CNTs	DPV	0.13 nM	10 nM–1 μM	[109]
Monkeypox	dsDNA	CRISPR-CS	DPV	9.5 × 10^−20^ M	10^−21^ M–10^−12^ M	[113]
Dengue	DENV-1 RNA	CRISPR/Cas13a	ACV	0.78 fM	5 fM–50 nM	[114]
Malaria	PfHRP2	Aptamers	SWV	3.73 nM	-	[115]

**Table 3:** Summary of electrochemical sensors used for inflammatory disease detection in this chapter.

Inflammatory Diseases	Biomarker	Materials	Detection Methods	LOD	Range	Ref.
Sepsis	CRP LPS	LPS aptamers CRP antibodies	CA	LPS (0.343 pg/mL) CRP (0.05 μg/mL)	LPS (0.5–1000 pg/mL) CRP (0.1–20 μg/mL)	[122]
Hepatitis B	HBV-DNA	BN-CDs	ECL	18.08 aM	100 aM–1 nM	[125]
Hepatitis C	HCV gene	S-BN QDs	ECL	0.17 pmoL/L	5 pmoL/L–1 nmoL/L	[127]
RA	IL-6	AuNPs/graphene	CA	0.42 pg/mL	0.97–250 pg/mL	[129]
Acute myocarditis	miR-721	MOF@Au@G-triplex/hemin nanozyme	CA	0.25 fM	0.5 fM–1 nM	[132]
-	CRP	AuNPs@C-ZIF_67_	DPV	0.44 pg/mL	10 pg/mL–10 μg/mL	[134]
-	IFN-*γ*	AgNCs	LSV	1.7 pg/mL	5–1000 pg/mL 1–10 ng/mL	[130]
-	IL-6	Ab-SPA-Cys-AuNPs-THI-CMWCNTs	SWV	2.87 pg/mL	0.01–800 ng/mL	[138]
-	IL-6	NMC@AuNPs	DPV	0.14 pg/mL	0.5–1200 pg/mL	[139]

**Table 4:** Summary of electrochemical sensors used for neurodegenerative disease detection in this chapter.

Neurodegenerative Diseases	Biomarker	Materials	Detection Methods	LOD	Range	Ref.
AD	Aβ oligomers	Au-VG/CC	DPV	3.5 pM	10–2200 pM	[143]
AD	Tau ApoE4 Amyloid-β miRNA-101	Gold nanodendrites/PDMS mini-pillar	SWV	Tau (5.91 × 10^−11^ mg/mL) ApoE4 (7.14 × 10^−11^ mg/mL) Amyloid-β (8.6 × 10^−12^ mg/mL) miRNA-101 (91.4 pM)	10^−10^–10^−7^	[144]
PD	α-synuclein oligomer	Mg^2+^-DNAzyme exhibit	DPV	0.57 fg/mL	1 fg/mL–10 pg/mL	[147]
PD	α-syn	BET-[BMIm]	PDECL	63 aM	100.0 aM–10.0 fM	[148]
-	NEFL IL-6	CAb/SA-poly-HRP 80	CA	NEFL (5.22 pg/mL) IL-6 (3.69 pg/mL)	NEFL (5.22–1000 pg/mL) IL-6 (3.69–1000 pg/mL)	[149]
-	DA	MHA	Amperometry	1.4 pM	0.01–1000 nM	[151]

## Data Availability

Not applicable.
